# Efficient Dicer processing of virus-derived double-stranded RNAs and its modulation by RIG-I-like receptor LGP2

**DOI:** 10.1371/journal.ppat.1009790

**Published:** 2021-08-03

**Authors:** Yuqiang Zhang, Yan Xu, Yunpeng Dai, Zhe Li, Jiaxing Wang, Zhi Ye, Yanxin Ren, Hua Wang, Wan-xiang Li, Jinfeng Lu, Shou-Wei Ding, Yang Li

**Affiliations:** 1 State Key Laboratory of Genetic Engineering, School of Life Sciences, Fudan University, Shanghai, China; 2 Department of Microbiology & Plant Pathology, University of California, Riverside, California, United States of America; 3 CAS Key Laboratory of Animal Ecology and Conservation Biology, Institute of Zoology, Chinese Academy of Sciences, Beijing, China; Emory University, UNITED STATES

## Abstract

The interferon-regulated antiviral responses are essential for the induction of both innate and adaptive immunity in mammals. Production of virus-derived small-interfering RNAs (vsiRNAs) to restrict virus infection by RNA interference (RNAi) is a recently identified mammalian immune response to several RNA viruses, which cause important human diseases such as influenza and Zika virus. However, little is known about Dicer processing of viral double-stranded RNA replicative intermediates (dsRNA-vRIs) in mammalian somatic cells. Here we show that infected somatic cells produced more influenza vsiRNAs than cellular microRNAs when both were produced by human Dicer expressed *de novo*, indicating that dsRNA-vRIs are not poor Dicer substrates as previously proposed according to *in vitro* Dicer processing of synthetic long dsRNA. We report the first evidence both for canonical vsiRNA production during wild-type Nodamura virus infection and direct vsiRNA sequestration by its RNAi suppressor protein B2 in two strains of suckling mice. Moreover, Sindbis virus (SINV) accumulation *in vivo* was decreased by prior production of SINV-targeting vsiRNAs triggered by infection and increased by heterologous expression of B2 *in cis* from SINV genome, indicating an antiviral function for the induced RNAi response. These findings reveal that unlike artificial long dsRNA, dsRNA-vRIs made during authentic infection of mature somatic cells are efficiently processed by Dicer into vsiRNAs to direct antiviral RNAi. Interestingly, Dicer processing of dsRNA-vRIs into vsiRNAs was inhibited by LGP2 (laboratory of genetics and physiology 2), which was encoded by an interferon-stimulated gene (ISG) shown recently to inhibit Dicer processing of artificial long dsRNA in cell culture. Our work thus further suggests negative modulation of antiviral RNAi by a known ISG from the interferon response.

## Author summary

The function and mechanism of the interferon-regulated antiviral responses have been extensively characterized. Recent studies have demonstrated induction of antiviral RNA interference (RNAi) in somatic cells against several mammalian RNA viruses rendered incapable of RNAi suppression. However, little is known about Dicer-mediated production of virus-derived small-interfering RNAs (vsiRNAs) in these cells active in the type I interferon response. Here we show that the dsRNA precursors of influenza vsiRNAs were processed more efficiently than cellular precursor microRNA hairpins by wild-type human Dicer expressed *de novo* in Dicer-knockout somatic cells. We found that infection of two strains of suckling mice with wild-type Nodamura virus (NoV) was associated with production of silencing-active vsiRNAs and direct sequestration of duplex vsiRNAs by its RNAi suppressor protein B2. Our findings from *in vivo* infection with Sindbis virus recombinants expressing NoV B2 or carrying a vsiRNA-targeted insert provide evidence for an antiviral function of the induced RNAi response. Interestingly, NoV infection induces expression of RIG-I-like receptor LGP2 to inhibit vsiRNA biogenesis and promote virulent infection in suckling mice. Our findings together reveal efficient Dicer processing of vsiRNA precursors in interferon-competent somatic cells and suckling mice in contrast to synthetic long dsRNA examined previously by *in vitro* dicing.

## Introduction

Dicer enzymes in the RNase III family mediate the biogenesis of microRNAs (miRNAs) and small interfering RNAs (siRNAs) in plants and animals [1,2]. Mature miRNAs are produced by Dicer from hairpin precursor miRNAs (pre-miRNAs) to repress the translation of the target mRNAs [3,4]. However, siRNAs are processed from long double-stranded RNA (dsRNA) to initiate RNA interference (RNAi), defined as specific slicing of complementary RNAs by an Argonaute protein (AGO) in RNA-induced silencing complex (RISC) [1,5]. The RNAi pathway functions as a potent antiviral immunity in plants and invertebrates because these hosts produce highly abundant virus-derived siRNAs (vsiRNAs) by Dicer from viral dsRNA precursors to guide RISC-dependent clearance of virus RNAs [6]. In counter defense, plant and insect viruses have evolved viral suppressors of RNAi (VSRs) to block various steps in the antiviral RNAi pathway [6–8].

In vertebrate animals, the type I interferon (IFN) response is a major first line of defense against virus infection before the activation of adaptive immunity [9,10]. The IFN antiviral response is frequently initiated by cytoplasmic sensing of viral RNA ligands by retinoic acid-inducible gene I (RIG-I) or melanoma differentiation factor 5 (MDA5). Upon RNA binding, these RIG-I-like receptors (RLRs) interact with mitochondrial antiviral-signaling protein (MAVS, also known as VISA, IPS-1 or Cardif) to activate RLR signal transduction, leading to transcriptional induction of the genes encoding type I IFN and other genes in the nucleus [11]. Binding of the type I IFN by IFN-α/β receptor (IFNAR) on cell surface then activates an intracellular signaling cascade to drive the expression of hundreds of IFN-stimulated genes (ISGs). Some ISGs encode virus restriction factors whereas other ISG factors such as 2’-5’ oligoadenylate synthetases and dsRNA-dependent protein kinase R (PKR) inhibit cell growth by inducing global RNA degradation and protein translation shutdown, respectively [11]. LGP2 (laboratory of genetics and physiology 2) is the third member of RLRs, but lacks the amino-terminal domains conserved in RIG-I and MDA5 necessary for independent signal-transducing activity. Although not essential for the induction of the IFN response, LGP2 can modulate antiviral defense by promoting MDA5-mediated responses or acting as an inhibitor of RIG-I signaling [12–19].

Recent studies have provided evidence for the induction of the antiviral RNAi response in mammals [20–28]. These studies, including ours, have demonstrated production of abundant vsiRNAs predominantly 22 nucleotides (nt) long during the infection of undifferentiated and differentiated cells as well as mice with positive- or negative-strand RNA viruses [23–30]. Notably, Nodamura virus (NoV), influenza A virus (IAV), human enterovirus 71 (HEV71), and dengue virus-2 (DNV2) from 4 distinct RNA virus families encode structurally unrelated VSRs necessary for infection and active to suppress Dicer processing of the cognate viral dsRNA replicative intermediates (dsRNA-vRIs) into vsiRNAs [23–25,27,29].

Plants and most invertebrate species encode two or more Dicer genes with at least one member being dispensable for miRNA biogenesis and dedicated instead to processing long dsRNA into siRNA and antiviral RNAi [6,31–33]. In contrast, mammals encode a single Dicer [1], which recognizes pre-miRNAs as more efficient substrates than long dsRNA in *in vitro* dicing assays [34,35]. Moreover, because of the induction of cell growth inhibition and cell death in IFN-competent differentiated cells, artificial long dsRNA has been shown to trigger RNAi only in pluripotent embryonic stem cells (ESCs) and embryonic carcinoma cells defective in canonical IFN signaling [36–40]. Recent genetic studies also support an antagonistic role of the IFN response to RNAi since long dsRNA induces RNAi in differentiated cells after PKR knockout or inactivation of the type I IFN response by removal of *MAVS* or *IFNAR* [41,42]. Consistently, *LGP2* has recently been identified as an ISG factor to block long dsRNA-induced RNAi in mouse embryonic fibroblasts (MEFs) by inhibiting Dicer processing of long dsRNA into siRNAs [43].

As shown in synthetic RNAi induced by long dsRNA or short hairpin RNA, mammalian vsiRNAs are processed from dsRNA-vRIs by Dicer to direct AGO2-dependent antiviral RNAi [23–25,27,29,44]. In contrast to long dsRNA, however, abundant vsiRNAs are processed from dsRNA-vRIs made by viral RNA-dependent RNA polymerase (RdRP) during infection in several commonly used lines of differentiated cells and/or mice with mutant viruses, including NoV, IAV, HEV71 and DNV2, rendered defective in the expression or the activity of the cognate VSR, but remained competent in the induction of the IFN response [23,25,27,29,30]. All of the 4 validated VSRs are dsRNA-binding proteins and suppress Dicer processing of long dsRNA into siRNAs *in vitro*. However, a dominant population of vsiRNAs becomes undetectable after infection with any of these viruses expressing a functional VSR, which is thus similar to previous deep sequencing profiling of total small RNAs in mature cells infected with a range of wild type RNA viruses [45–50]. Although two wild type RNA viruse induce production of abundant vsiRNAs in undifferentiated cells [24,26], production of vsiRNAs in the IFN-defective mouse ESCs induced by NoV infection is potently suppressed by the cognate VSR protein B2 [24], suggesting widespread suppression of vsiRNA biogenesis during mammalian virus infection [6,20]. Moreover, *MAVS* knockout in MEFs activates synthetic RNAi induced by artificial long dsRNA without enhancing RNAi-mediated antiviral defense shown to be active in wildtype MEFs [42,44]. A receent study also indicates that antiviral RNAi requires AGO4, which is dispensable for RNA slicing by synthetic siRNAs [51].

Several key questions remain unresolved in mammalian antiviral RNAi induced in differentiated cells and *in vivo*. First, it is unclear whether vsiRNA precursors are inherently poor substrates of human Dicer in differentiated cells as it is widely known for long dsRNA dicing *in vitro* [34,35]. Second, it is also unclear whether canonical vsiRNAs are produced during infection of differentiated cells or *in vivo* by wild type viruses with or without a validated VSR although it is known that natural infection of MEFs by wild type NoV and IAV is inhibited by RNAi [29,44]. Third, recent studies have provided evidence for vsiRNA-dependent RNA degradation [25,44]. However, it remains unknown whether the vsiRNAs can mediate specific inhibition of virus accumulation *in vivo*. In this work, we designed and performed experiments to address these unresolved questions in mammalian antiviral RNAi. Moreover, we investigated whether the recently identified inhibitor of artificial long dsRNA Dicer processing, LGP2, is also active against vsiRNA production *in vitro* and *in vivo*. Our findings reveal key aspects of antiviral RNAi induced by RNA virus infection that are different or similar to the induction of RNAi by artificial long dsRNA in mammalian somatic cells.

## Results

### Human Dicer recognized and processed more viral dsRNA replication products than cellular pre-miRNA hairpins in somatic cells

We first developed an experimental system to compare the accumulation of vsiRNAs and cellular miRNAs processed from their precursors in differentiated human cells. We and others have previously demonstrated Dicer-mediated biogenesis of the influenza vsiRNAs from dsRNA-vRI precursors in human 293T cells infected with PR8/delNS1, a VSR-NS1 (non-structural protein 1) deletion mutant of IAV-strain PR8 [29,30]. In this work, we infected the Dicer-deficient (NoDice) human 293T cells [52] with PR8/delNS1 6 hours post-transfection with a human Dicer (hDcr)-expressing plasmid and sequenced the total small RNAs in the infected cells 24 hours post-infection. NoDice cells accumulate little miRNAs [52]. Thus, it became possible in our experimental system to compare the relative accumulation levels of vsiRNAs and cellular miRNAs processed from their respective precursors by the Dicer enzyme expressed *de novo* in the same sequenced small RNA library.

**Fig 1:**
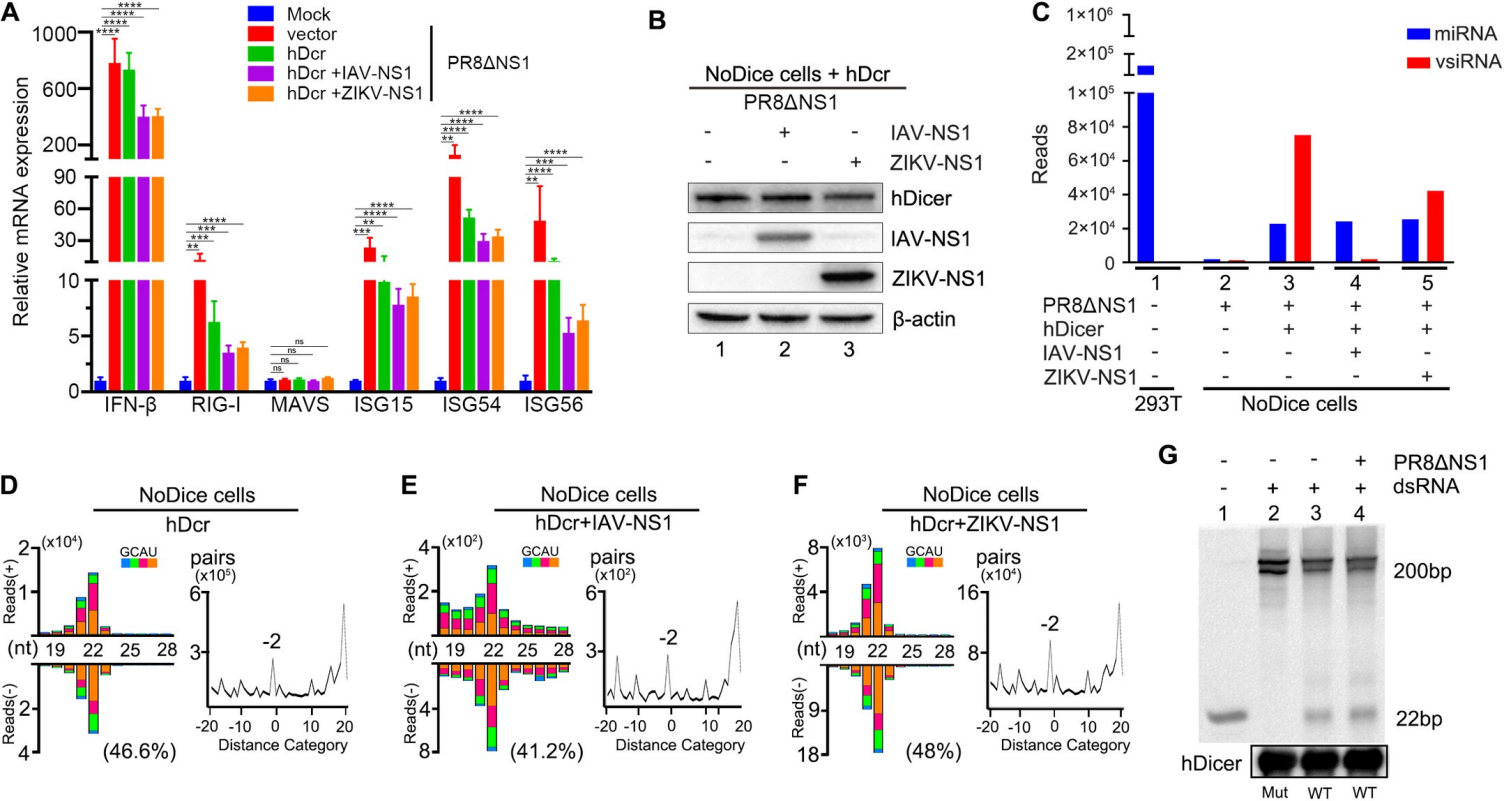
Efficient Dicer processing of IAV derived-dsRNA in human 293T cells active in type I IFN response. A. Induction of type I IFN response in NoDice cells. NoDice cells were mock-inoculated or infected with PR8/delNS1 at 6 hours post-transfection with the plasmid vector, an hDcr-expressing plasmid alone or together another plasmid to express IAV-NS1 or ZIKV-NS1. The accumulation levels of IFN-β, RIG-I, MAVS, ISG15, ISG54, and ISG56 mRNAs were determined by RT-qPCR at 24 hours post-infection. Each experiment was repeated at least three times independently and error bars indicate the standard deviation. The mRNA level of mock-inoculated cells without plasmid transfection was set as 1. ** indicates p<0.01, *** indicates p<0.001, **** indicates p<0.0001 (Student’s t-test). ns indicates no significance. B. Western blotting detection of the ectopically expressed human Dicer and/or IAV-NS1 /hDcr+ZIKV-NS1 as described in (A). Endogenous β-actin was detected as a loading control. C. Relative abundance of cellular miRNAs and IAV-derived 21- to 23-nt small RNAs per million total 18- to 28-nt reads in the individual small RNA libraries prepared from the NoDice cells at 24 hours post-infection as described in (A). D-F. Size distribution of IAV-derived 18- to 28-nt small RNAs (left) and duplex pattern of 22-nt IAV vsiRNAs (right) in the 3 libraries (no. 3, 4 and 5) presented in (C) from PR8/delNS1-infected NoDice cells ectopically expressing hDcr alone (D) or with IAV-NS1 (E) or ZIKV-NS1 (F). Data information: Reads were shown as per million total 18- to 28-nt reads. The 5’ terminal nucleotide of 18- to 28-nt viral small RNAs was indicated by color and 1U % of 21- to 23-nt vsiRNA given in parenthesis. The “-2” peak corresponded to the pair of canonical 22-nt vsiRNAs with a 20-nt duplex region plus 2-nt 3’ overhangs, calculated by an algorithm described previously counting pairs of complementary 22-nt vsiRNAs in each distance category (in nucleotides) between 5’ and 3’ ends of each pair. Length (nt) indicates x-axis for size distribution. Distance category indicates x-axis for duplex pattern. G. In vitro dicing of a synthetic 200 bp dsRNA by FLAG-tagged wild-type (lanes 3 & 4) or mutant (lane 2) human Dicer immune-precipitated from NoDice cells with or without PR8/delNS1. The Dicer substrate and product RNAs were fractionated by 15% PAGE and detected by GelRed staining. Lane 1: 22bp marker. Each experiment was repeated at least three times independently with one represented image shown.

We found that infection with the VSR-deficient IAV induced expression of ISGs, including *IFNβ* and *RIG-I*, in NoDice cells with or without the ectopic expression of hDcr (Fig 1A), indicating induction of the type I IFN response by PR8/delNS1 infection as expected [53]. Ectopic expression of hDcr was confirmed by Western blotting (Fig 1B). Deep sequencing verified rescue of the severely impaired miRNA biogenesis in NoDice cells by the ectopically expressed hDcr (Fig 1C and S1 Table) as previously reported [52]. Also as described previously [29], we detected hDcr-dependent production of a typical population of predominantly 22-nt influenza vsiRNAs in NoDice cells (Fig 1D–1F). By comparison, however, the influenza vsiRNAs made during the one-day infection were 229.5% more abundant than the total human miRNAs accumulated in NoDice cells (Fig 1C, lane 3 and S1 Table). These influenza vsiRNAs had an approximately equal ratio of positive and negative strands, and contained a dominant population of 22-nt vsiRNA duplexes with 2-nt 3’ overhangs, indicating that they were Dicer products processed from dsRNA-vRIs of the 8 negative-strand genomic RNAs (Figs 1D–1F and S1). Thus, the influenza dsRNA-vRIs synthesized by the viral RdRP were recognized as the main substrate of hDcr in the infected human cells with an active type I IFN response.

We further sequenced the total small RNAs in PR8/delNS1-infected NoDice cells that co-expressed hDcr with the non-structural protein 1 (NS1) encoded by either IAV or Zika virus (ZIKV). The two viral NS1 proteins antagonize the IFN response by distinct mechanisms [53,54] and IAV NS1 exhibits an additional activity to suppress vsiRNA biogenesis [29,55]. The expression of both NS1 proteins was confirmed by Western blotting (Fig 1B). We found that expression of neither IFN antagonistic protein had an obvious effect on the abundance of the total human miRNAs made by hDcr in the infected NoDice cells (Fig 1C, lanes 3, 4 and 5, left; S1 Table). A typical population of the influenza vsiRNAs was produced by hDcr in the infected NoDice cells expressing NS1 of either IAV or ZIKV (Fig 1E and 1F). We noted that the virus genome distribution patterns of vsiRNAs were similar in all three libraries (S1 Fig). However, expression of both viral NS1 proteins reduced the abundance of the influenza vsiRNAs with the lowest abundant vsiRNAs detected in cells expressing IAV NS1 (Fig 1C, lanes 3, 4 and 5, right), consistent with its known VSR activity [29,55]. Interestingly, we found that the influenza vsiRNAs were 66.2% more abundant than host miRNAs in the NoDice cells expressing NS1 of ZIKV (Fig 1C, lane 5, right and S1 Table). Compared to cellular pre-miRNA hairpins, therefore, the influenza dsRNA-vRIs remained as the dominant substrates of human Dicer in the infected cells when type-I IFN production is inhibited by ZIKV NS1 shown previously at the step of TBK1 complex formation [54].

To verify the dicing activity of the ectopically expressed hDcr, we assayed *in vitro* processing of 200-nt dsRNA by FLAG-tagged hDcr co-immunoprecipitated from NoDice cells with or without induction of the IFN response by PR8/delNS1 infection. As shown in Fig 1G, we detected efficient processing of the dsRNA into 22-nt siRNAs by wild-type hDcr, but not a Dicer mutant in which the catalytic sites were mutated (Fig 1G, lanes 2 and 3). Importantly, we observed no obvious differences in the processing of the long dsRNA by hDcr purified from either the mock- or PR8/delNS1-infected cells (Fig 1C, lanes 3 and 4), indicating that hDcr remained active after the induction of the IFN response. Together, our results demonstrate that when the biogenesis of both vsiRNAs and cellular miRNAs was mediated by the Dicer enzyme expressed *de novo*, viral dsRNA replicative intermediates served as the more dominant substrates than pre-miRNAs in the IFN-competent human cells.

### Production and direct VSR sequestration of canonical duplex vsiRNAs in two strains of mice infected with a wild type RNA virus

We next examined the hypothesis that VSR-expressing wild type virus infection *in vivo* also triggers production of canonical vsiRNAs, which, however, are not readily visible from deep sequencing of total small RNAs due to the presence of abundant viral RNA degradation products. It is known that vsiRNAs are selectively loaded into RISC and the VSR-B2 protein encoded by NoV and the closely related Flock house virus exhibits duplex siRNA-binding activity *in vitro* [6,56–59]. Thus, we initially searched for the presence of *in vivo* vsiRNAs by sequencing total small RNAs both before and after co-immunoprecipitation (IP) with B2-specific antibodies or a pan-Argonaute antibody from NoV-infected BALB/c suckling mice at 3 days post-inoculation (dpi).

**Fig 2:**
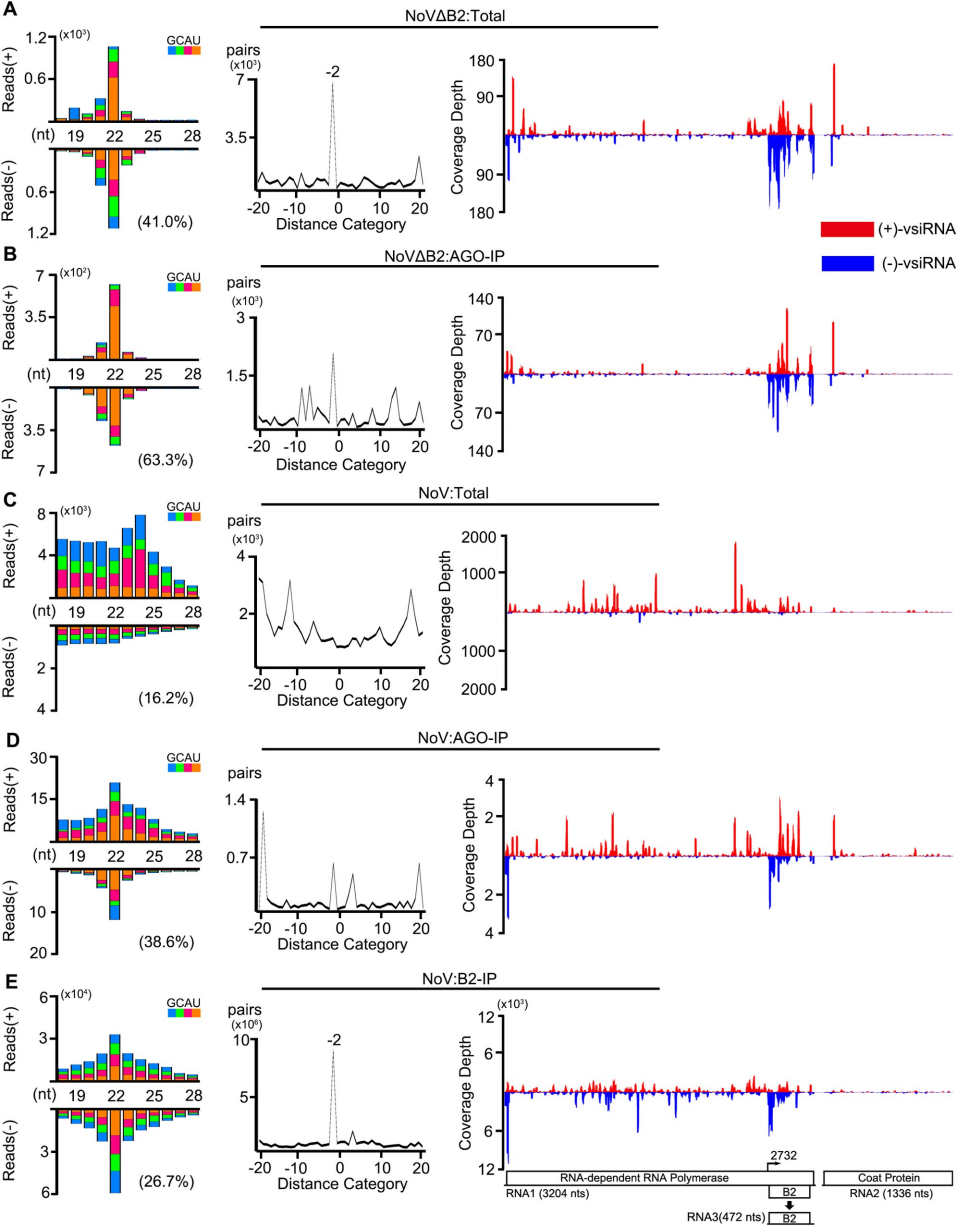
NoV infection *in vivo* induces production of abundant canonical duplex vsiRNAs sequestered in VSR-B2 protein complex. A-E. Size distribution of 18- to 28-nt virus-derived small RNAs (left), duplex pattern of the 22-nt vsiRNAs (middle) and the distribution of 21- to 23-nt vsiRNAs along the viral genomic RNAs 1 and 2 sequenced from BALB/c suckling mice infected with NoVΔB2 (A, B) or NoV (C, D, E) at 3 dpi, either with or without co-immunoprecipitation (co-IP) by antibodies specific to mouse AGOs (B, D) or the viral B2 protein (E). Data information: Same as in Fig 1.

We detected abundant vsiRNAs in the Argonaute precipitants from NoVΔB2-infected suckling mice in the control experiments. Similar to the total vsiRNAs, the most dominant size class in Argonaute-bound vsiRNAs of both polarities was 22-nt (Fig 2A and 2B, left panels). The distribution patterns of vsiRNA hot spots on the two positive-strand genomic RNAs of NoV were similar between the total and Argonaute-bound populations (Fig 2A and 2B, right panels), suggesting that the vsiRNAs produced by mice in response to infection are loaded proportionally into the Argonaute complexes. However, Argonaute-bound vsiRNAs exhibited an increased enrichment for vsiRNAs in the size range of 21- to 23-nt (92.8%) and for 1U vsiRNAs (63.3%), which were 85.9% and 41.0% for the total vsiRNAs, respectively (Fig 2A and 2B and S1 Table). Similar to our recent findings from the mutant mice defective in adaptive immunity [44], these findings indicate that the vsiRNAs produced in IFN-competent wild type mice are *in vivo* loaded into Argonaute complexes with strong selection for 21- to 23-nt 1U vsiRNAs. As a further indicative measure of selective loading [45], the population of 22-nt duplex vsiRNAs with 2-nt overhangs (shown by the -2 peak in Fig 2A and 2B, middle panel) was less dominant for Argonaute-bound vsiRNAs than the total vsiRNAs.

As described previously [23], the total vsRNAs sequenced from NoV-infected mice were highly abundant, but showed no preference in the size range of Dicer products and were mostly positive strands (Fig 2C). Notably, B2 immunoprecipitants from the same NoV-infected mice contained highly abundant vsRNAs with approximately equal ratios of positive and negative strands and the 22-nt as the most dominant species for both polarities. Moreover, B2-bound 22-nt vsRNAs were overwhelmingly enriched for 22-nt canonical siRNA duplexes with 2-nt 3′ overhangs without 1U preference (Fig 2E). Argonaute-bound vsiRNAs were also detectable in NoV-infected mice, but were approximately 25-fold less abundant in NoV-infected mice than those in NoVΔB2-infected mice (Fig 2B and 2D and S1 Table). These findings reveal Dicer-mediated production of vsiRNAs from viral dsRNA replicative intermediates in NoV-infected mice, which were sequestered by B2 before Argonaute loading.

**Fig 3:**
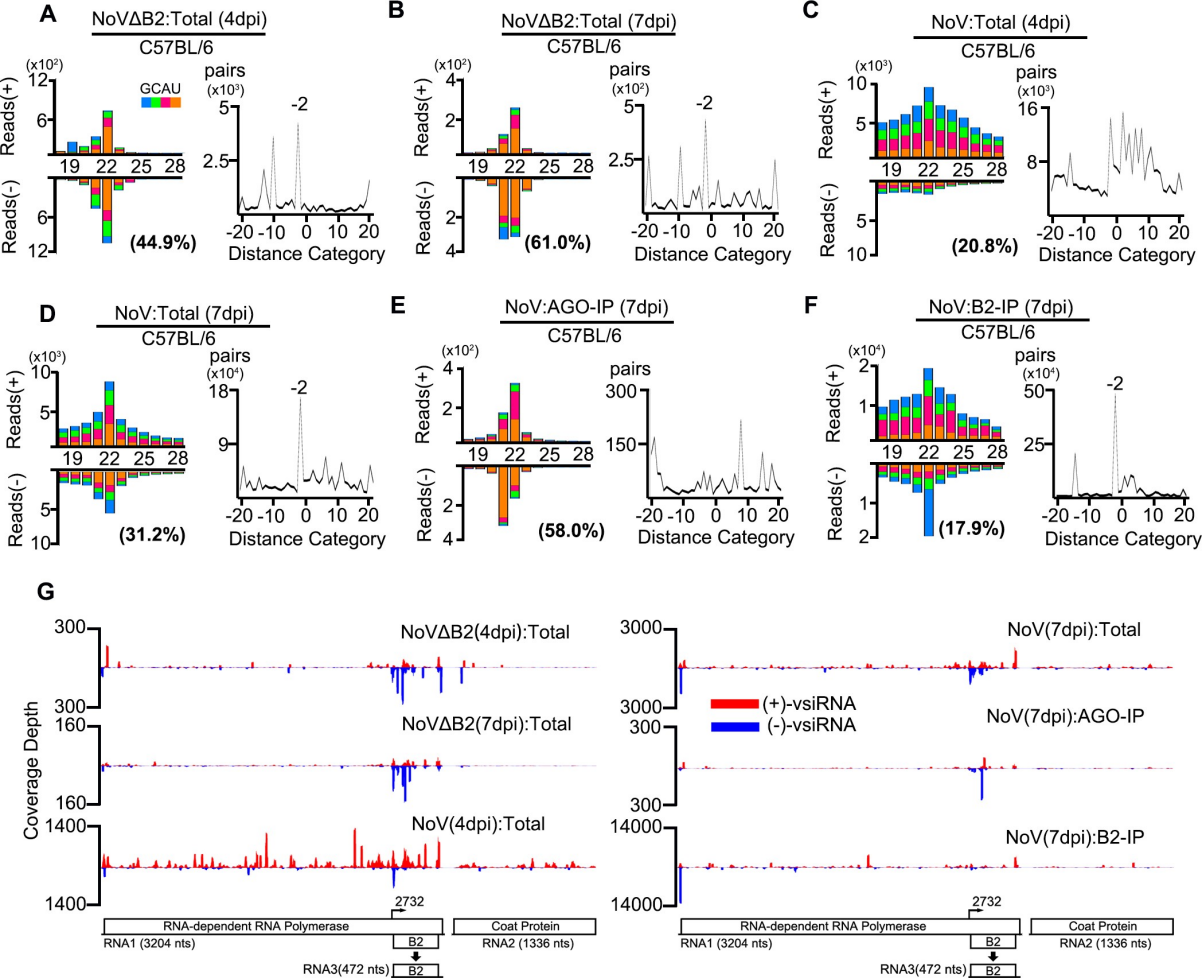
Abundant vsiRNAs and their sequestration by VSR-B2 in C57BL/6 mice infected with wild-type NoV. A-F. Size distribution of 18- to 28-nt virus-derived small RNAs (left) and duplex pattern of the 22-nt vsiRNAs (right) sequenced from NoVΔB2 and NoV-infected C57BL/6 suckling mice at 4 (A, C) or 7 dpi (B, D), either with or without co-IP by antibodies specific to mouse AGOs proteins (E) or the viral B2 protein (F). G. The distribution of 21- to 23-nt vsiRNAs along the viral genomic RNAs 1 and 2 from the six libraries presented from (A) to (F). Reads are shown as per million total 18- to 28-nt reads. Data information: Same as in Fig 1.

C57BL/6 suckling mice developed a delayed lethal disease after NoV infection compared to BALB/c mice (see below), which allowed us to sequence total and co-immunoprecipitated small RNAs from suckling mice at both 4 and 7 dpi (S2 Fig). As in BALB/c mice (Fig 2), we observed vsiRNA production and the interference of vsiRNA biogenesis in C57BL/6 mice 4 days after infection with NoVΔB2 and NoV, respectively (Fig 3). The vsiRNAs remained abundant in NoVΔB2-infected mice at 7 dpi (Fig 3B). In contrast to the earlier time points in BALB/c and C57BL/6 (Figs 2C and 3C), however, NoV-infected mice at 7 dpi accumulated highly abundant vsRNAs exhibiting clearly visible properties of vsiRNAs, including a dominant peak at 22 nt for both strands and strong enrichment for 22-nt vsiRNA duplexes with 2-nt 3’ overhangs (Fig 3D). The percentage of 21- to 23-nt vsiRNAs relative to total mature miRNAs from NoV-infected C57BL/6 mice was able to reach 7.1% (S1 Table). The 22-nt canonical vsiRNA duplexes accumulated to high levels in B2 complexes from NoV-infected C57BL/6 mice (Fig 3F), providing further evidence for *in vivo* B2 sequestration of duplex vsiRNAs. Nevertheless, the Argonaute immunoprecipitants from NoV-infected C57BL/6 mice at 7 dpi contained much more abundant 1U vsiRNAs than those from NoV-infected BALB/c mice at 3 dpi (Figs 2D and 3E). Together, our findings provide the first evidence for the *in vivo* production and sequestration of canonical duplex vsiRNAs by VSR-B2 in response to the infection with a VSR-expressing wild type virus.

### *In cis* expression of NoV VSR-B2 from Sindbis virus interfered with vsiRNA biogenesis and enhanced virus accumulation *in vivo*

**Fig 4:**
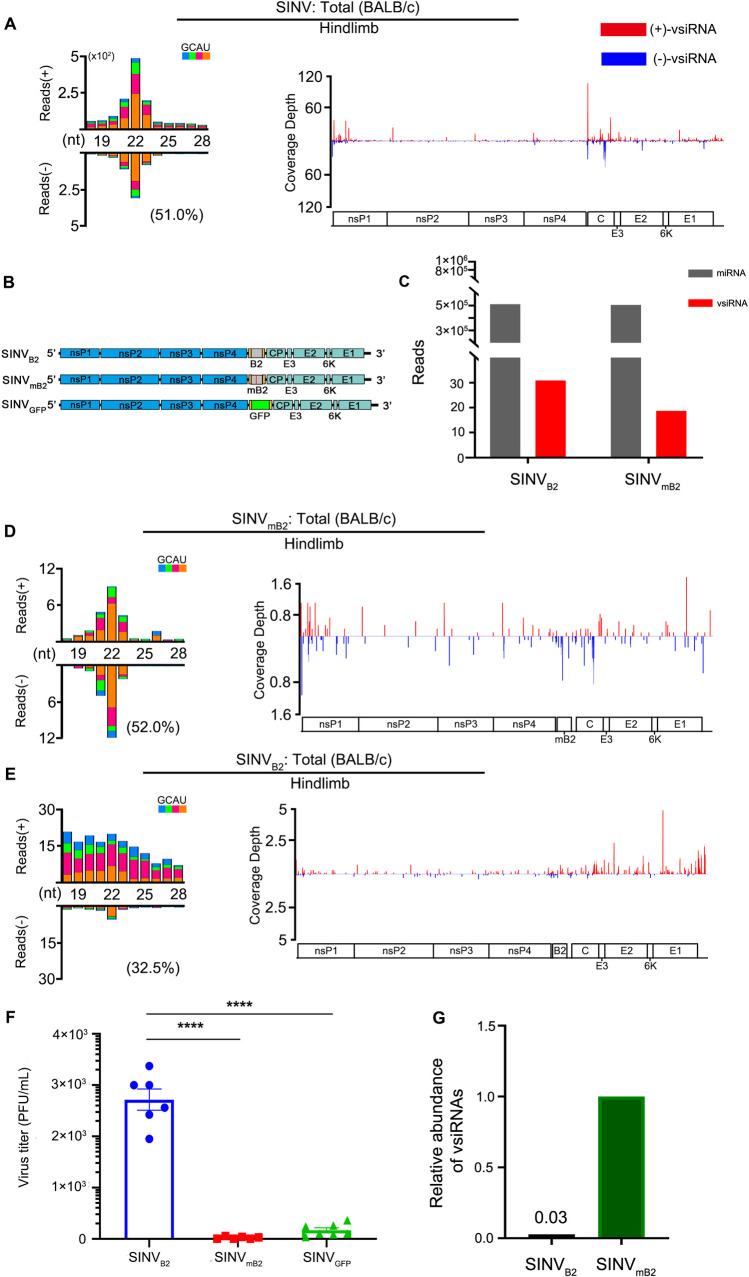
*In vivo* function of a heterologous VSR expressed *in cis* from Sindbis virus genome. A, D, and E: Virus-derived small RNAs produced by BALB/c suckling mice in response to the infection with wild-type (A) or recombinant SINV expressing wild-type (E) or mutant (D) VSR-B2 of NoV by intraperitoneal injection at 3 dpi. Size distribution of 18- to 28-nt virus-derived small RNAs (left) and the distribution of 21- to 23-nt vsiRNAs along the viral genomic RNA (right) were shown as per million total mature miRNAs. B. The genomic structure of SINV_B2_, SINV_mB2_, and SINV_GFP_. C. Relative abundance of mature miRNAs and 21- to 23-nt vsiRNAs sequenced from BALB/c suckling mice infected with SINVB2 or SINVmB2 at 3 dpi. Reads counts were shown as per million total 18- to 28-nt reads. F. Viral titer (PFU/ml) in the hindlimb of 6 to 7 individual BALB/c suckling mice infected with recombinant SINV_B2_, SINV_mB2_, or SINV_GFP_ at 3dpi was measured by a standard plaque assay and normalized by tissue mass. **** indicates p<0.0001, Student’s t-test. G. Relative abundance of cellular miRNAs and 21- to 23-nt vsiRNAs sequenced from BALB/c suckling mice infected with SINV_B2_ or SINV_mB2_ at 3 dpi. Reads counts were normalized by both per million total 18- to 28-nt reads and virus accumulation levels determined by RT-qPCR.

We further determined whether the B2 protein of NoV acts as a functional VSR when expressed from a heterologous positive-strand RNA virus, Sindbis virus (SINV), recently documented to trigger production of vsiRNAs in infected brain tissues of suckling mice after intracranial inoculation [28]. We first cloned and sequenced the total small RNAs from hind limb muscle tissue of BALB/c suckling mice inoculated by intraperitoneal injection. The profile of SINV-derived small RNAs exhibited the “hallmark” of vsiRNAs, with the 22-nt vsiRNAs as the most abundant population (Fig 4A), suggesting lack of strong viral suppression of vsiRNA biogenesis in wild-type SINV-infected mice as has been shown in mosquitoes [60].

We next constructed three SINV recombinants for mouse infection (Fig 4B). We found that the hind limb muscle tissue of BALB/c mice also accumulated a typical population of vsiRNAs after intraperitoneal injection with SINV_mB2_, a recombinant SINV engineered to express a non-functional B2 mutant (mB2) with a single amino acid substitution (Fig 4D). In contrast to infection with SINV or SINV_mB2_, deep sequencing of small RNAs from mice infected with SINV_B2_ engineered to express wild-type B2, revealed a population of mostly positive-strand vsRNAs without the size preference of vsiRNAs (Fig 4E), indicating that *in cis* expression of VSR-B2 interfered with the biogenesis of vsiRNAs during SINV_B2_ infection. Furthermore, viral plaque assays showed that SINV_B2_ replicated to significantly enhanced levels in the infected mice than either SINV_mB2_ or SINV_GFP_, engineered to express GFP from the same genomic position of SINV as B2 (Figs 4F and S3). Although total 21–23 nt vsRNA reads in mice infected with SINV_B2_ were slightly more abundant than those in SINV_mB2_-infected mice (Fig 4C), they were much less abundant in SINV_B2_-infected mice than SINV_mB_ -infected mice after normalization by viral accumulation (Fig 4G). These results indicate that *in cis* expression of a functional VSR-B2 from SINV genome interfered with the biogenesis of vsiRNAs and enhanced the accumulation of the heterologous virus in the infected suckling mice, suggesting *in vivo* inhibition of SINV infection by antiviral RNAi.

### Biological activity of mammalian vsiRNAs produced in the response to NoV infection *in vivo*

We next investigated whether *in vivo* production of vsiRNAs mediates specific inhibition of virus accumulation. Production of vsiRNAs confers resistance in plants against heterologous viruses engineered to contain a segment from the vaccinating virus [61]. Sequence specific degradation of chimeric reporter mRNAs has been observed in cultured cells induced to produce complementary vsiRNAs [25]. We recently took a similar approach to determine the homology-dependent viral RNA degradation *in vivo* since this strategy avoids the unintended consequences of the genetic manipulation of Dicer or Argonaute-2 on endogenous miRNAs and development [28,62]. In this work, we investigated whether production of SINV-targeting vsiRNAs in mice prior to SINV inoculation could enhance the inhibition of SINV infection.

**Fig 5:**
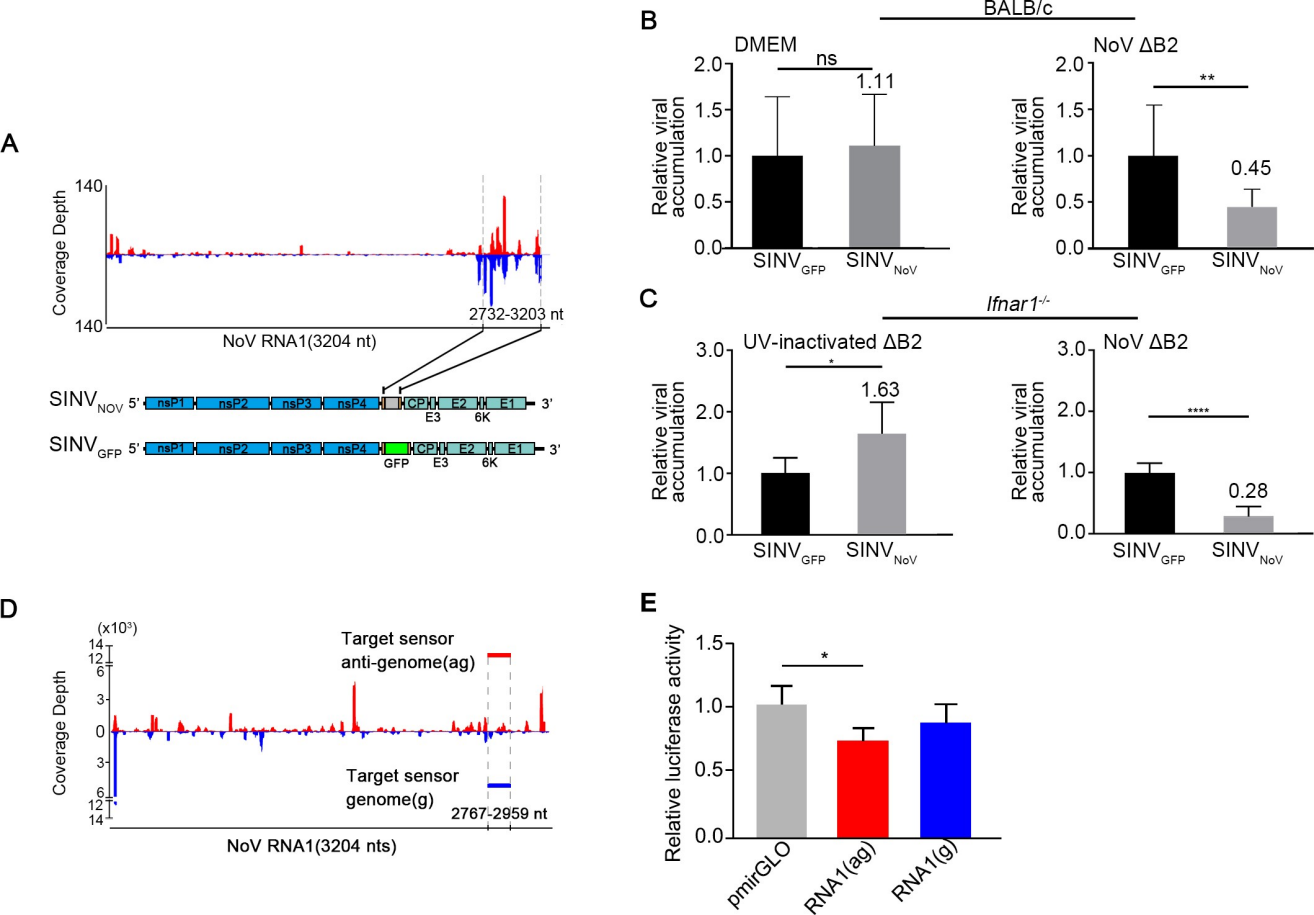
The viral siRNAs of NoV inducing homology-dependent viral RNA degradation. A. The genomic structure of a recombinant SINV (SINV_NoV_) carrying a segment of NoV RNA1 known to be targeted by high densities of vsiRNAs in NoVΔB2-infected mice shown at the top. Also shown below was the genomic structure of SINV_GFP_ introduced above. B. *In vivo* genomic RNA levels of SINV_NoV_ and SINV_GFP_ one day after challenge inoculation of NoVΔB2-infected (right) or DMEM mock-infected (left) BALB/c suckling mice (n = 7 per DMEM group, n = 10 per NoVΔB2 group). The viral genomic RNA accumulation level was determined by RT-qPCR amplification of the viral nsP2 coding region and normalized by endogenous actin mRNA with SINV_GFP_ level set as 1. Error bars represent SD. ** indicates p<0.01 (Student’s t-test). C. *In vivo* genomic RNA levels of SINV_NoV_ and SINV_GFP_ measured as in (B) one day after challenge inoculation of *Ifnar1*^*-/-*^ suckling mice (n = 5~7 per group) inoculated two days earlier with live (right) or UV-inactivated (left) NoVΔB2. Error bars represent SD. * indicates <0.05, **** indicates p<0.0001 (Student’s t-test). The viral RNA accumulation of SINV_GFP_ was set as 1. D. Diagram showing the genomic position of the 200-nt NoV sequence in the genome (g, blue) or anti-genome (ag, red) sense inserted into the 3′ untranslated region (UTR) of firefly luciferase reporter mRNA, to be targeted respectively by antisense and sense vsiRNAs extracted from VSR-B2 immuno-precipitants of NoV-infected C57BL/6 suckling mice. E. Relative luciferase activity of the control and chimeric reporter constructs with a 3’-UTR containing a NoV sequence targeted by the positive- or negative-strand vsiRNAs sequestered in VSR-B2 complex. Error bars indicate standard deviation of three replicates. Error bars represent SD. * indicates p<0.05 (Student’s t-test).

We constructed a recombinant Sindbis virus, SINV_NoV_, which contained an insert corresponding to a region of NoV genomic RNA1 targeted by high densities of vsiRNAs in NoVΔB2-infected mice (Fig 5A). It is predicted that SINV_NoV_, but not SINV_GFP_ used as a control, will be specifically targeted for RNAi by the vsiRNAs produced in the mice after infection with NoVΔB2. Indeed, we found that SINV_NoV_ accumulated to significantly lower levels than SINV_GFP_ in mice pre-inoculated with NoVΔB2, but not with buffer DMEM (Fig 5B). Infection with live NoVΔB2 also induced significant inhibition on the accumulation of SINV_NoV_ compared to that of SINV_GFP_ in type I IFN receptor knockout mice (*Ifnar1*^*-/-*^, Fig 5C), which are defective in the signaling by type I IFNs [63]. It appeared that NoVΔB2-induced suppression of SINV_NoV_ accumulation was more effective in *Ifnar1*^*-/-*^ mice than BALB/c mice (Fig 5B and 5C). For unknown reason, however, SINV_NoV_ replicated to higher levels than SINV_GFP_ in *Ifnar1*^*-/-*^ mice immunized with UV-inactivated NoVΔB2 (Fig 5C). We propose that prior production of SINV_NoV_-targeting vsiRNAs induced by NoVΔB2 immunization in BALB/c and *Ifnar1*^*-/-*^ suckling mice directed suppression of virus infection by RNAi in a mechanism similar to the sequence-specific protection from incoming RNA viruses in *Ifnar1*^*-/-*^ MEFs induced by transfection with long dsRNA [42].

We also investigated the silencing activity of the vsiRNAs produced in NoV-infected mice using an mRNA reporter approach similar to that described by Qiu *et al* (2017). A vsiRNA-targeted fragment of NoV RNA1 in sense or antisense orientation was inserted into the 3′ UTR of a dual luciferase reporter plasmid (Fig 5D). Human 293T cells were transfected with one of the reporter constructs together with the NoV-specific vsiRNAs extracted from B2 immunoprecipitants obtained from NoV-infected C57BL/6 suckling mice as described above (Fig 3F). Although we observed suppression of the luciferase reporter containing the NoV fragment in either orientation compared to the control 24 hours after transfection, the difference was statistically significant only for the luciferase reporter targeted by antisense vsiRNAs (Fig 5E), indicating gene silencing activity for the vsiRNAs made by C57BL/6 mice in response to NoV infection and sequestered by VSR B2.

### LGP2 inhibits Dicer-mediated biogenesis of vsiRNAs from dsRNA-vRIs

**Fig 6:**
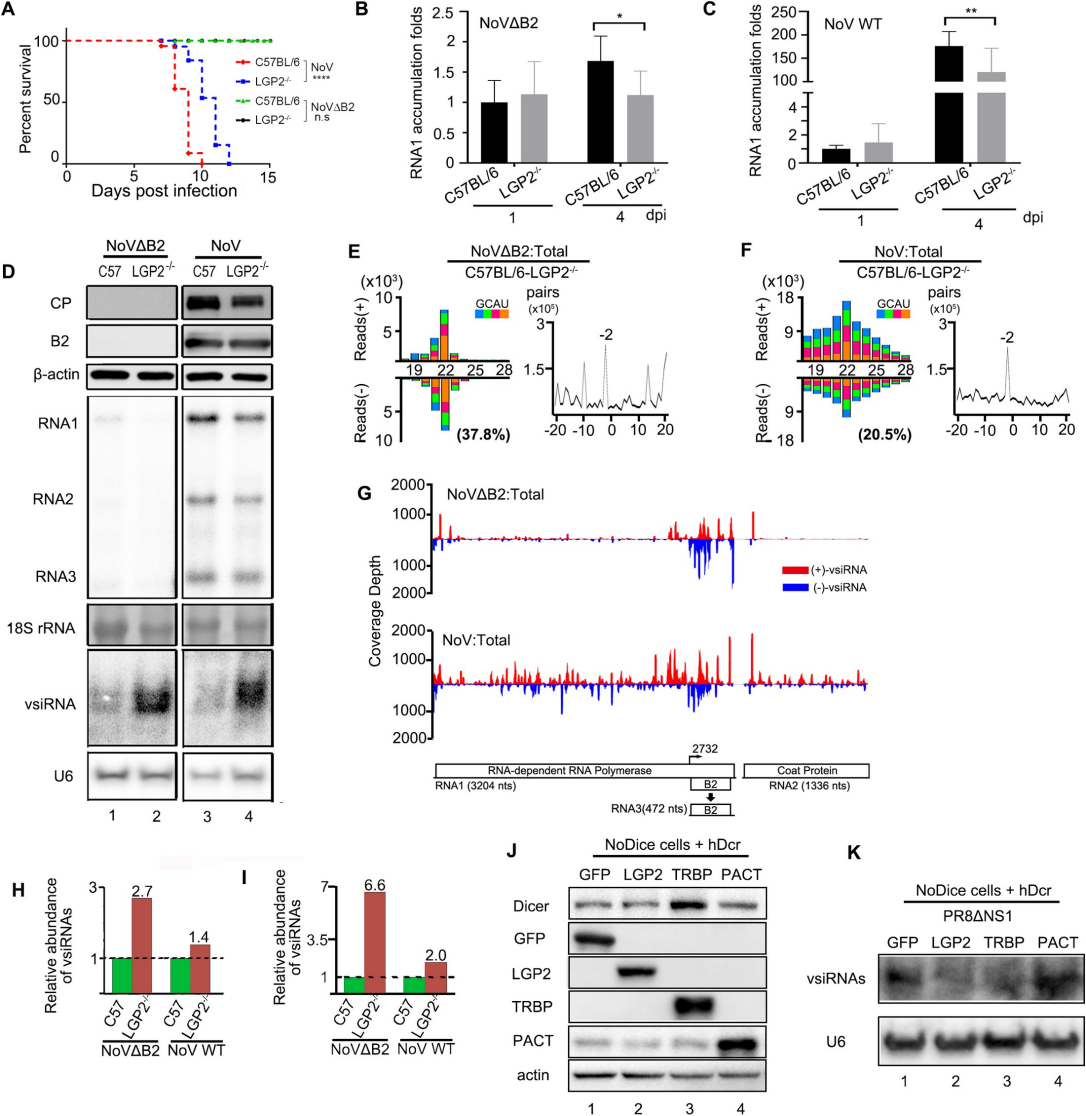
Enhanced production of vsiRNAs and reduced virulence of NoV in *Dhx58*^*-/-*^ suckling mice compared to parental C57BL / 6 mice. A. Survival curve of C57BL/6 and *Dhx58*^*-/-*^ suckling mice inoculated with WT NoV. Data analysis was performed using GraphPad Prism. n = 12~14. Log-rank (Mantel-Cox) test. **** indicates p<0.0001. B, C RNA1 accumulation level of NoVΔB2 (B) or WT NoV (C) measured by RT-qPCR from hindlimb of C57BL/6 or *Dhx58*^*-/-*^suckling mice at 1 or 4 dpi. The RNA1 level of NoVΔB2 infected C57BL/6 suckling mice at 1 dpi was set as 1. Error bars indicate standard deviation of three replicates. * indicates p<0.05, ** indicates p<0.01 (Student’s t-test). D. Western blotting detection (upper 3 panels) of the viral coat protein (CP) and B2 protein in C57BL/6 or *Dhx58*^*-/-*^ suckling mice infected with NoVΔB2 or NoV at 4 dpi. Detection of the endogenous β-actin was used as a loading control. Northern blotting detection (lower 4 panels) of viral genomic RNAs and vsiRNAs in C57BL/6 and *Dhx58*^*-/-*^ suckling mice inoculated with NoVΔB2 or NoV at 4 dpi. Detection of 18S rRNAs and U6 were used as loading controls. E, F. Size distribution 18- to 28-nt virus-derived small RNAs (left) and duplex pattern of the 22-nt vsiRNAs (right) from NoVΔB2 (E) or WT NoV (F) infected *Dhx58*^*-/-*^ suckling mice at 4 dpi. Reads shown as per million total mature miRNAs. The 5’ terminal nucleotide and 1U % of 21- to 23-nt vsiRNAs were indicated. G. The distribution of 21- to 23-nt vsiRNAs along the viral genomic RNAs 1 and 2 from the libraries of (E) and (F). H, I. Relative abundance of 21- to 23-nt vsiRNAs sequenced from C57BL/6 and *Dhx58*^*-/-*^ suckling mice inoculated with NoVΔB2 or NoV at 4dpi. Reads were normalized either by mature miRNAs only (H) or by both mature miRNAs and virus titer determined by RT-qPCR (I). J, K. Western blotting detection of GFP, LGP2, TRBP, and PACT proteins (J) and Northern blotting of vsiRNAs (K) in PR8/delNS1-infected NoDice cells ectopically co-expressing hDcr with GFP, LGP2, TRBP or PACT. Detection of the endogenous β-actin protein and U6 rRNA were used as loading controls. Each experiment was repeated at least three times independently.

Finally, we examined the role of LGP2 encoded by *DHX58* gene in NoV infection *in vivo*, which has recently been shown to block artificial long dsRNA-induced RNAi in *IFNAR* knockout MEFs by inhibiting Dicer processing of long dsRNA into siRNAs [43]. Both C57BL/6 suckling mice and *Dhx58*^*-/-*^ suckling mice on a pure C57BL/6 background [12] were infected with the same dose of NoV and NoVΔB2 by intraperitoneal injection. We monitored mouse survival and virus accumulation in the limb muscular tissues of the infected mice (Fig 6). NoVΔB2 induced no signs of disease in both genotypes of mice; however, *Dhx58*^*-/-*^ mice survived approximately 1 or 2 days longer than C57BL/6 mice after NoV infection and the difference in survival was statistically significant (Fig 6A). Northern and Western blotting as well as quantitative RT-PCR revealed that NoV accumulated to lower levels in *Dhx58*^*-/-*^ mice than wild-type mice at 4 dpi (Fig 6B–6D). Moreover, although NoVΔB2 was cleared in both genotypes of mice, *Dhx58*^*-/-*^ mice also supported lower levels of NoVΔB2 accumulation than C57BL/6 mice at 4 dpi (Fig 6B–6D). These results suggest that LGP2 expression enhanced the *in vivo* accumulation of both NoV and NoVΔB2.

The induction of *IFNβ*, *DHX58* and several other ISGs were verified in suckling mice after infection with either NoV or NoVΔB2 (S4–S6 Figs). Deep sequencing of small RNAs showed that whereas both the strand ratio and the size preference of the vsiRNAs were similar, the relative abundance of vsiRNAs triggered by NoVΔB2 infection was higher in *Dhx58*^*-/-*^ mice (2.7%) than in wild-type mice (1.0%) (Figs 3A and 6E and S1 Table). Northern blotting of small RNAs further confirmed that NoVΔB2 infection triggered an increased accumulation of vsiRNAs in *Dhx58*^*-/-*^ mice compared to wild-type mice even though NoVΔB2 replicated to lower levels in *Dhx58*^*-/-*^ mice (Fig 6D). Furthermore, several properties of vsiRNAs were readily visible for the total vsRNAs sequenced from NoV-infected *Dhx58*^*-/-*^ mice at 4 dpi (Fig 6F), unlike those from NoV-infected wild-type C57BL/6 mice at 4 dpi (Fig 3C). These included a dominant 22-nt peak for both the positive and negative strands, an increased abundance of the negative strands (37.4% vs 14.3% in wild-type mice), an increased preference in the size range of 21- to 23-nt (46.1% vs 39.1%), and strong enrichment of a single dominant population of 22-nt canonical vsiRNA duplexes with 2-nt 3’ overhangs (Figs 3C and 6F and S1 Table). Stronger signals in the size range of vsiRNAs were detectable by Northern blotting for RNA samples extracted from NoV-infected *Dhx58*^*-/-*^ mice than wild-type mice (Fig 6D). vsiRNAs were more abundant in *Dhx58*^*-/-*^ mice than C57BL/6 mice infected with either NoVΔB2 or WT NoV (Fig 6H), particularly after normalization by virus titer (Fig 6I). These results suggest that LGP2 inhibited Dicer processing of the dsRNA-vRIs into vsiRNAs during the *in vivo* infection with both NoV and NoVΔB2.

We further compared the production of the influenza vsiRNAs triggered by PR8/delNS1 infection in NoDice 293T cells expressing hDcr together with GFP or LGP2 (Fig 6J and 6K). Northern blotting revealed strong inhibition of the influenza vsiRNA accumulation by LGP2 compared to the control (Fig 6K). In this assay (Fig 6K), the biogenesis of the influenza vsiRNAs appeared to be suppressed by TAR-RNA binding protein (TRBP), but not the protein activator of PKR (PACT), both of which are dsRNA-binding proteins involved in Dicer-mediated miRNA biogenesis and formation of the RISC complex. Our findings indicate that LGP2 inhibits Dicer-mediated biogenesis of vsiRNAs from dsRNA-vRIs, revealing another similarity between RNAi induced by dsRNA-vRIs and artificial long dsRNA [43].

## Discussion

Results from this work reveal several new insights into the induction and suppression of Dicer-mediated production of mammalian vsiRNAs during infection of mammalian somatic cells. We found that influenza vsiRNA precursors became the more dominant Dicer substrates than pre-miRNA hairpins in mature human cells when both vsiRNAs and cellular miRNAs were processed by human Dicer expressed *de novo*. Unlike synthetic long dsRNA identified as less efficient Dicer substrates than pre-miRNA hairpins from previous *in vitro* biochemical studies [34,35], therefore, our findings show that the dsRNA-vRIs made by viral RdRP are not inherently poor substrates of human Dicer in the infected cells.

Our results provide the first evidence that canonical vsiRNAs processed from dsRNA-vRIs were produced in two strains of IFN-competent suckling mice after wild-type virus infection. Similar to previous studies on cell culture infections with a range of wild-type viruses [20–28,45–50], we were not able to find canonical vsiRNAs from NoV-infected mice at 3 or 4 dpi by deep sequencing of total small RNAs. However, the hallmarks of vsiRNAs were clearly visible when the same samples were enriched by VSR-B2 co-immunoprecipitation before sequencing. These VSR-sequestered vsiRNAs made *in vivo* directed gene silencing of a luciferase reporter in human cells, providing the first example for *in vivo* sequestration of functional vsiRNAs by a mammalian VSR. Interestingly, canonical vsiRNAs became readily detectable by deep sequencing without prior VSR-B2 co-immunoprecipitation in NoV-infected wild-type C57BL/6 mice by 7 dpi. In these infected mice, abundant vsiRNAs were loaded into RISC and the Argonaute-bound vsiRNAs exhibited strong selection for 1U vsiRNAs of 21 to 23 nucleotides in length, similarly to those found in NoVΔB2-infected wild-type suckling mice, cultured cells [29] or mutant adult mice defective in the adaptive immunity reported recently by Han et al [44]when this manuscript was under preparation. These findings support our earlier conclusion that NoV-infected mice produce RNA slicing-competent vsiRNAs-RISC [44]. Our results suggest that examining *in vivo* infection at different time points or removing non-specific small RNAs by Argonaute or VSR co-immunoprecipitation may facilitate detection of vsiRNAs by deep sequencing.

Our characterization of suckling mouse infection with SINV recombinants expressing VSR-B2 (SINV_B2_) or a segment of NoV genomic RNA 1 (SINV_NoV_) indicated an *in vivo* function of the vsiRNA response induced by dsRNA-vRIs. Similar to the infection of mosquitoes active in antiviral RNAi [60], we found that SINV_B2_ accumulated to significantly higher levels in BALB/c mice than both SINV_GFP_ and SINV_mB2_, which expressed GFP or a mutant B2 protein unable to interfere with the biogenesis of the vsiRNAs. By contrast, SINV_NoV_ replicated to significantly lower levels than SINV_GFP_ in the suckling mice accumulating abundant vsiRNAs to target the inserted NoV sequence due to prior infection/immunization with NoVΔB2. Together, our findings suggest an *in vivo* antiviral activity of the vsiRNAs triggered by infection with SINV recombinants or NoVΔB2.

Finally, we found that over-expression of the ISG factor LGP2 in human 293T cells potently inhbited Dicer processing of the influenza dsRNA-vRIs into vsiRNAs. Thus, LGP2 inhibits Dicer processing of both dsRNA-vRIs in 293T cells shown in this work and artificial long dsRNA reported previously in IFN-deficient MEFs [43]. As expected, infection of suckling mice with either NoV or NoVΔB2 induced expression of the LGP2 gene *Dhx58*. Notably, both viruses replicated to lower levels, but triggered production of vsiRNAs at higher levels in *Dhx58*^*-/-*^ mice than wild-type mice. These findings suggest negative modulation of antiviral RNAi by LGP2, which may explain why immunization with NoVΔB2 induced weaker suppression of SINV_NoV_ accumulation in wild-type mice than *Ifnar1*^*-/-*^ mice defective in the IFN-mediated induction of *Dhx58*. As noted previously [43], our findings also support the functional similarity of LGP2 with one of the three RLRs encoded by the single-Dicer nematode *Caenorhabditis elegans*, Dicer-related helicase 2 (DRH-2), which suppresses antiviral RNAi and lacks the N-terminal domain conserved in DRH-1 and DRH-3 [64,65]. Together, our findings further highlight the differences and similarities in the induction of RNAi by the dsRNA-vRIs made by viral RdRP and artificial long dsRNA in mammalian somatic cells.

## Materials and methods

### Ethics statement

All animal experiments in China were performed in Fudan University of China and under the guidelines of the Institutional Animal Care and Use Committee. Animals in USA were housed in the Animal Resources Facility according to the guidelines described under the federal Animal Welfare Regulations Act and the procedures were approved by the Institutional Animal Care and Use Committee at the University of California, Riverside.

### Viruses and cell culture

Wildtype Nodamura virus (NoV) and mutant NoVΔB2 strains used in this study were described previously [23]. Influenza A virus (IAV), PR8/delNS1 (NS1 deletion mutant) was gift from Dr. A. Garcıa-Sastre and Dr. P. Palese. Sindbis virus (SINV) was rescued from the plasmids as previously described [28]. The NoDice Human embryonic kidney (293T) cell line and its parental 293T cell line were gifts from from Dr. B. Cullen. African green monkey kidney epithelial cells (Vero) were purchased from the American Type Culture Collection (ATCC). Cells were cultured in Dulbecco’s modified Eagle’s medium (DMEM) containing 10% fetal bovine serum at 37°C with 5% CO_2_.

### Animals

Experimental animals, including BALB/c, C57BL/6 and *Ifnar1*^*-/-*^, were purchased from Jackson labs (Bar Harbor, ME), Shanghai SLAC laboratory Animal Co., Ltd., and Cyagen Biosciences. *Dhx58*^*-/-*^ mice was gift from Dr. Michael Gale, Jr., University of Washington.

### Plasmids

The sequences encoding NS1 of IAV-WSN or ZIKV were amplified by reverse transcription-polymerase chain reaction (RT-PCR) using specific primers containing the restriction enzyme sites and cloned into pcDNA3.1(-) vector to generate plasmids expressing NS1 protein of IAV and ZIKV. The expression plasmids for human Dicer (hDcr) were purchased from Addgene (cat. # 19873, #41584 and #41585). The plasmids expressing EGFP and human LGP2, TRBP, PACT were constructed into pEF-BOS entry vector using standard molecular cloning technique. The pTE/5’2J/GFP and pTE/5’2J were gifts from Dr. C.M. Rice. To obtain recombination SINV virus expressing B2 protein of NoV and sequence of RNA1, we constructed the plasmids of pTE/5’2J/B2, pTE/5’2J/mB2 and pTE/5’2J/NoV by ligating PCR products of NoV B2, mB2 (mutant B2, R59Q) and NoV RNA1 (2732–3203 nt) flanked by *Xba* I and *Apa* I into the MCS of pTE/5’2J, which was used to rescue recombinant SINV virus of SINV_B2_, SINV_mB2_, SINV_NOV_ as previously described [28]. The pmirGLO Dual-Luciferase miRNA Target Expression Vector was purchased from promega, which encode the firefly luciferase and *Renilla* luciferase genes. The pmirGLO-RNA1(ag)/RNA1(g) reporters were designed by cloning part of the viral genome sequence NoV (anti-genome and genome of 2767–2959 nt respectively) into pmirGLO plasmid using *Xba* I sites and ClonExpress II One Step Cloning Kit (Vazyme). Primers used are listed in S2 Table. All plasmids were confirmed by sequencing.

### Western and Northern blotting analyses

Western and Northern blotting analysis was performed as described previously [29,66]. Antibodies to IAV-NS1, NoV B2 protein and coat protein (Capsid) were described previously [23,66], antibodies to Dicer, LGP2, GFP, TRBP and PACT (Santa Cruz Biotechnology, sc 136979, sc 134668, sc 9996, sc 100909, sc 81569), ZIKV-NS1 (GeneTex) are sourced from commercial suppliers. Probes used for Northern blotting to detect miRNA are listed in S2 Table and to measure vsRNAs were shown previously [23,29].

### Cell culture transfection and infection

NoDice 293T cells (2.5 × 10^6^ per 6 cm plate) were seeded one day before transfection. Cells were transfected with different recombination plasmids, including 8 μg plasmid encoding hDicer and mock or 4 μg following plasmids, pcDNA-IAV-NS1, pcDNA-ZIKV-NS1, pEF-BOS-GFP, pEF-BOS-LGP2, pEF-BOS-TRBP and pEF-BOS-PACT using Lipofectamine 2000 (Thermo) according to the supplier’s recommended protocol and then infected by PR8/delNS1 (MOI = 1) at six hours after transfection. The infected cells were harvested for the extraction of total protein and RNA using TRIzol 24 hours after infection.

### Preparation of long dsRNA

The dsRNAs used for *in vitro* assay of Dicer activity were prepared as described before [42]. Briefly, to generate (+)-sense RNA GFP and (-)-sense RNA GFP, two PCR fragments were amplified from pcDNA3.1-GFP plasmid (Promega) corresponding to the first 200 nt of GFP. The PCR products were purified using QIAquick PCR purification kit (Qiagen), and *in vitro* transcription (IVT) with T7 RNA polymerase (T7 MEGAscript kit, Ambion) was performed according to the manufacturer’s instructions. The dsRNAs were generated by mixing an equal volume of (+)-sense IVT RNA and (-)-sense IVT RNA, incubation at 95°C for 10 min followed by cool down to RT. The dsRNAs were purified and separated on a 2% agarose gel to verify size and integrity.

### *In vitro* assay of Dicer activity

Human Flag-Dicer and Flag-mutant-Dicer in pCAGGS (Addgene #41584 and #41585) were transfected into NoDice 293T cells using Lipofectamine 2000. Mutant Dicer construct? NoDice 293T cells were seeded in a 6-well plate at a density of 5 × 10^5^ per well one day before transfection. NoDice 293T cells were transfected with 3 μg plasmids. 48 hours after transfection, hDcr-KO 293T cells were infected by PR8/delNS1 (MOI = 1) or DMEM and the infected cells were lysed in cell lysis buffer (CST) 24 hours after infection. The Flag-Dicer was retrieved using FLAG beads (GenScript) according to the manufacturer’s instructions. For dicing assays, Flag-Dicer was incubated with 50 nM dsRNA in dicing buffer [30 mM Tris pH 6.8, 50 mM NaCl, 3 mM MgCl2, 5% glycerol, 1 mM DTT, RNAsin (Promega)] for 1 h at 37°C followed by Trizol purification. The RNA was resuspended in formamide sample buffer without xylene blue (47.5% formamide, 0.01% SDS, 0.01% bromophenol blue, 0.5 mM EDTA), loaded onto a 15% TBE-Urea gel and visualised by GelRed staining.

### NoV and SINV infection

NoV or NoVΔB2 preparations shown to contain 7x10^6^ copies of genomic RNA1 from the titrated set of stocks was inoculated to each of suckling mice of 6 to 8 days old after birth by intraperitoneal injection (i.p.) as described previously [23]. 50 PFU of SINV was inoculated to each of suckling mice of 6 to 8 days old after birth by intraperitoneal injection. Total RNAs and proteins were purified from the hind limb muscle tissues of infected suckling mice at different time.

### Co-immunoprecipitation

Hind limb muscle tissue of suckling mice infection with NoV or NoVΔB2 were co-immunoprecipitated (co-IP) by anti-pan Argonaute (Ago) antibody (Millipore, Billerica, MA) and by B2 antibodies as described. Briefly, 3 μg of rabbit or mouse IgG and 15 μl of protein A/G PLUS-Agarose beads (Santa Cruz Biotechnology) were incubated with 100 μg of muscle tissue lysates in 1 ml RIPA. After pre-cleared, 3 μg of anti-pan Ago, B2 antibodies, or controls IgG antibody immobilized to protein A/G PLUS-Agarose beads were added for 2 hours at 4°C. The total RNAs obtained from precipitated complexes were used for the construction of small RNA libraries as described [23]. Due to the extremely low RNA content in the controls IgG antibody immunoprecipitants, these samples were not included in subsequent library construction and sequencing.

### Viral plaque assays

In brief, hindlimb muscles were isolated from infected or mock infected suckling mice. After homogenization, 1mL DMEM were added to per 50mg tissue. The tissue homogenates were used for plague assay. Vero cells were plated at a density of 5.0 × 10^5^ cells/ well in 3 ml DMEM, 10% FBS, on 6-well plates and incubated at 37°C, 5% CO2 atmosphere. On the next day, 1ml of 10-fold gradient dilutions of supernatants were added to each well. After incubation of 1 h, supernatants were discarded and the cells were overlaid with 5mL 1×MEM containing 0.5% agarose in each well. After incubation of 2 days, the agarose gels were removed, and cell layer was stained with commassie brilliant blue. Virus content of the supernatants was calculated as plaque forming units (PFU)/ml.

### *In vivo* recombinant SINV reporter experiments

For BALB/c suckling mice, seven-day-old mice were inoculated by i.p. with NoVΔB2 (shown to contain 7x10^6^ copies of genomic RNA1) or with the same volume of DMEM (mock). Two days after inoculation, the mice were infected by i.p. with SINV_GFP_ or SINV_NoV_ viruses of 500 PFU. Each group of suckling mice were euthanized one day after SINV infection to determine virus titers in the hind limb tissue by RT-qPCR. For *Ifnar1*^*-/-*^ mice, seven-day-old mice were inoculated by i.p. with NoVΔB2 (shown to contain 7x10^6^ copies of genomic RNA1) or with UV-inactivation NoVΔB2. Two days after inoculation, *Ifnar1*^*-/-*^ mice were infected by i.p. with SINV_GFP_ or SINV_NoV_ viruses of 500 PFU and euthanized one day after SINV infection to determine virus titers in the hind limb tissue by RT-qPCR.

### Luciferase reporter assay

293T cells were seeded in a 12-well plate at a density of 5 × 10^5^ per plate one day before transfection. Cells were transfected with 1 μg of pmirGLO-Control vector or pmirGLO-RNA1(ag)/RNA1(g) vector, together with 0.3 μg of total small RNA extracted from B2 immunoprecipitants described above, with *Trans*IT-TKO Transfection Reagent (Mirus). Luciferase activity was analyzed using the Dual-Luciferase Reporter Assay System (Cat. #E1910, Promega) and measured on Fluoreskan Ascent FL (Thermo Scientific) after 24 hours transfection, and the firefly luciferase values were divided by *Renilla* luciferase values to normalize for transfection efficiency.

### RT-qPCR

Total RNA was extracted using TRIzol reagent following the manufacturer’s instructions (Thermo Fisher Scientific). 1 μg of RNA was reverse transcribed using PrimeScript RT Reagent Kit with gDNA Eraser (Takara), and qPCR was performed using iQ SYBR Green Supermix (Bio-rad). Reactions were carried out using CFX-Connect Real-Time System (Bio-rad). Relative expression values were calculated using the ΔΔCt method normalized by β-actin housekeeping gene. All primers used for RT–qPCR were listed in S2 Table.

### Statistical analysis

All statistical analysis of RT-qPCR data were performed by GraphPad Prism 8 using unpaired Student’s t test. All experiments were repeated independently at least three times. A P value of <0.05 was considered statistically significant.

### RNA-seq and data analysis

Total RNA was extracted from infected and mock hind limb muscle tissue using TRIzol reagent. The integrity of the purified RNA was analyzed by the Agilent 2200 Electrophoresis Bioanalyzer System (Agilent Technologies). Enrichment of poly (A)-RNA preparation for RNA Sequencing was performed using NEBNext Poly (A) mRNA Magnetic Isolation Module (NEB) kit. The cDNA libraries were constructed for each pooled RNA sample using the NEBNext Ultra Directional RNA Library Prep Kit for Illumina according to the manufacturer’s instructions. The products were purified and enriched by PCR to create the final cDNA libraries and quantified by Agilent2200. The tagged cDNA libraries were pooled in equal ratio and used for 150 bp paired-end sequencing in a single lane of the Illumina HiSeqXTen. Clean reads were obtained from the raw reads by removing the adaptor sequences, reads with > 5% ambiguous bases (noted as N) and low-quality reads containing more than 20 percent of bases with qualities of < 20. The clean reads were then aligned to mouse genome (version: GRCh38 NCBI) using the hisat2 [67]. HTSeq software was used to count the number of reads mapped to each gene [68]. Differential expression analysis of any two groups was performed using the DESeq package [69] and the differentially expressed genes between samples were identified after the significant analysis and false discovery rate (FDR) analysis under the following criteria [70]: (1) fold change > 2 or < 0.5; (2) FDR< 0.05. Gene Ontology (GO) analysis was applied to analyze the primary functions of the differentially expressed genes according to the GO [71]. Fisher’s exact test was applied to identify the significant GO categories and FDR was used to correct the p-values.

### Construction of small RNA libraries

RNA preparations in this study were used for the construction of small RNA libraries by the method that depends on the 5’ monophosphate of small RNAs as described previously with the TruSeq Small RNA Sample Preparation Kit of Illumina (San Diego, CA) [29].

### Deep sequencing and bioinformatic analysis of small RNAs

Libraries of small RNAs were cloned from the RNA samples (mice n = 3; cell samples, repeat once) and sequenced by Illumina HiSeq 2000/2500. 19 libraries in total were sequenced from this work (S1 Table). Mapping was done by Bowtie 1.1.2 with perfect match. Subsequent bioinformatics analysis of virus-derived small RNAs was carried out using in-house Perl scripts as described previously [29]. Pairs of complementary 22-nt vsiRNAs in each library with different base-pairing lengths were computed using a previously described algorithm [23]. The reference sequences used in this study are either identical with those described previously or as listed below:

1. NoV RNAs 1 and 2: AF174533.1 and AF174534.1
2. NoVΔB2 RNAs 1 and 2: the same as NoV except for 3 substitutions in RNA1: U2745C, U2754C, C2757G.
3. PR8/delNS1: Obtained from A/Puerto Rico/8/34 (H1N1) (PR8-WT) by deleting nucleotides 57 to 528 in the NS segment. The sequence of PR8-WT: AF389115.1, AF389116.1, AF389117.1, AF389118.1, AF389119.1, AF389120.1, AF389121.1 and AF389122.1.
4. SINV: j02363.1
5. Mature miRNAs and miRNA precursors: miRBase 21 (http://www.mirbase.org/).

## Supporting information

S1 Fig Genomic coverage depth of viral small RNAs, related to Fig 1. A-C. PR8/delNS1-infected NoDice 293T cells ectopically expressing hDcr (A), hDcr+IAV-NS1 (B) and hDcr+ZIKV-NS1 (C). Genomic coverage depth of each nucleotide position by 21- to 23-nt vsiRNAs sequenced from RNA above. Reads are shown as per million total 18- to 28-nt reads.
(TIF)

S2 Fig Relative viral accumulation of NoV-infected C57BL/6 mice. A. RNA1 accumulation level of WT NoV or NoVΔB2 measured by RT-qPCR from hind limb of C57BL/6 suckling mice at 7 dpi (n = 5 per group). The RNA1 level of NoVΔB2 infected C57BL/6 suckling mice was set as 1. B. Expression level of NoV capsid and B2 protein measured by Western blotting. Actin were used as a loading control.
(TIF)

S3 Fig Relative viral accumulation of SINV_B2_, SINV_mB2_, SINV_GFP_. The genomic structure of SINV_B2_, SINV_mB2_ or SINV_GFP_ (right) and relative viral accumulation determined by RT-qPCR from hindlimb of BALB/c suckling mice infected with SINV_B2_, SINV_mB2_ or SINV_GFP_ at 3dpi. n = 6~7 per group. Error bars represent SD. * indicates p<0.05 (Student’s t-test). The viral RNA accumulation of SINV_GFP_ was set as 1.
(TIF)

S4 Fig Characteristic of IFN inducing in C57BL/6 mice with NoV infection. A, B. Expression levels of IFN (A) and RNAi (B) related genes in C57BL/6 suckling mice with NoV infection at 7dpi. All data were measured as the mean ± SD of three independent experiments. Asterisks indicate a significant difference level compared to control (Student’s t-test, *p<0.05, **p<0.01, ***p<0.001, ****p<0.0001).
(TIF)

S5 Fig Differential expression of IFN pathway related gene in NoV infected BALB/c mice. A, B. Volcano plots showing demonstrate false discovery rate (FDR) and fold-change (FC) of gene expression levels determined by RNAseq comparing BALB/c suckling mice inoculated by NoVΔB2 vs Mock (A), WT NoV vs Mock (B). Genes up-regulated (fold change > 2 and FDR< 0.05) are indicated in red and those down-regulated (fold change < 0.5 and FDR< 0.05) are indicated in blue. C. Differential expression of IFN pathway related genes from mRNA-seq data of NoV or NoVΔB2 inoculated BALB/c suckling mice at 3 dpi. Fold changes (FC) of 2 or 0.5 (|log_2_FC| = 1) are indicated by dotted lines. The log_2_FC was taken as 20 when the ratio of experimental group and mock group tended to be infinite due to a small denominator.
(TIF)

S6 Fig Differential expression of IFN pathway related gene in NoV infected C57BL/6 mice. A, B. Volcano plots showing demonstrate false discovery rate (FDR) and fold-change (FC) of gene expression levels determined by RNAseq comparing C57BL/6 suckling mice inoculated by NoVΔB2 vs Mock (A), WT NoV vs Mock (B). Genes up-regulated (fold change > 2 and FDR< 0.05) are indicated in red and those down-regulated (fold change < 0.5 and FDR< 0.05) are indicated in blue. C. Differential expression of IFN pathway related genes from mRNA-seq data of NoV or NoVΔB2 inoculated C57BL/6 suckling mice at 3 dpi. Fold changes (FC) of 2 or 0.5 (|log_2_FC| = 1) are indicated by dotted lines. The log_2_FC was taken as 20 when the ratio of experimental group and mock group tended to be infinite due to a small denominator.
(TIF)

S1 Table Contents and properties of the small RNA libraries sequenced. (DOCX)

S2 Table Primers, Related to Experimental Procedures. (DOCX)
