# Supplementary figures and images for: Efficient Dicer processing of virus-derived double-stranded RNAs and its modulation by RIG-I-like receptor LGP2
Source: PLoS Pathog. 2021 Aug 3;17(8):e1009790. doi: 10.1371/journal.ppat.1009790 (PMC8362961; doi:10.1371/journal.ppat.1009790)

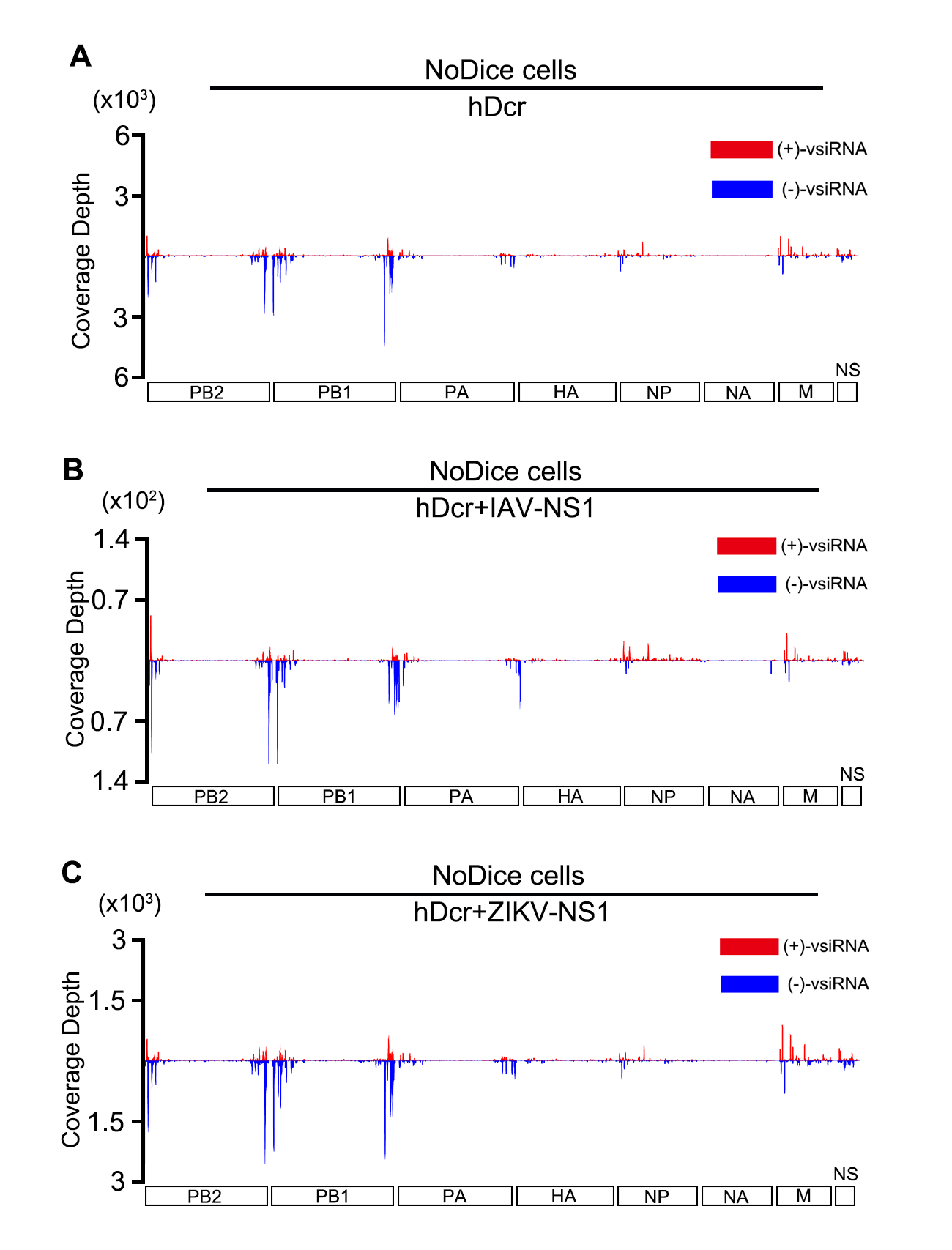

Supplement: S1 Fig — A-C. PR8/delNS1-infected NoDice 293T cells ectopically expressing hDcr (A), hDcr+IAV-NS1 (B) and hDcr+ZIKV-NS1 (C). Genomic coverage depth of each nucleotide position by 21- to 23-nt vsiRNAs sequenced from RNA above. Reads are shown as per million total 18- to 28-nt reads. (TIF) [file ppat.1009790.s001.tif]

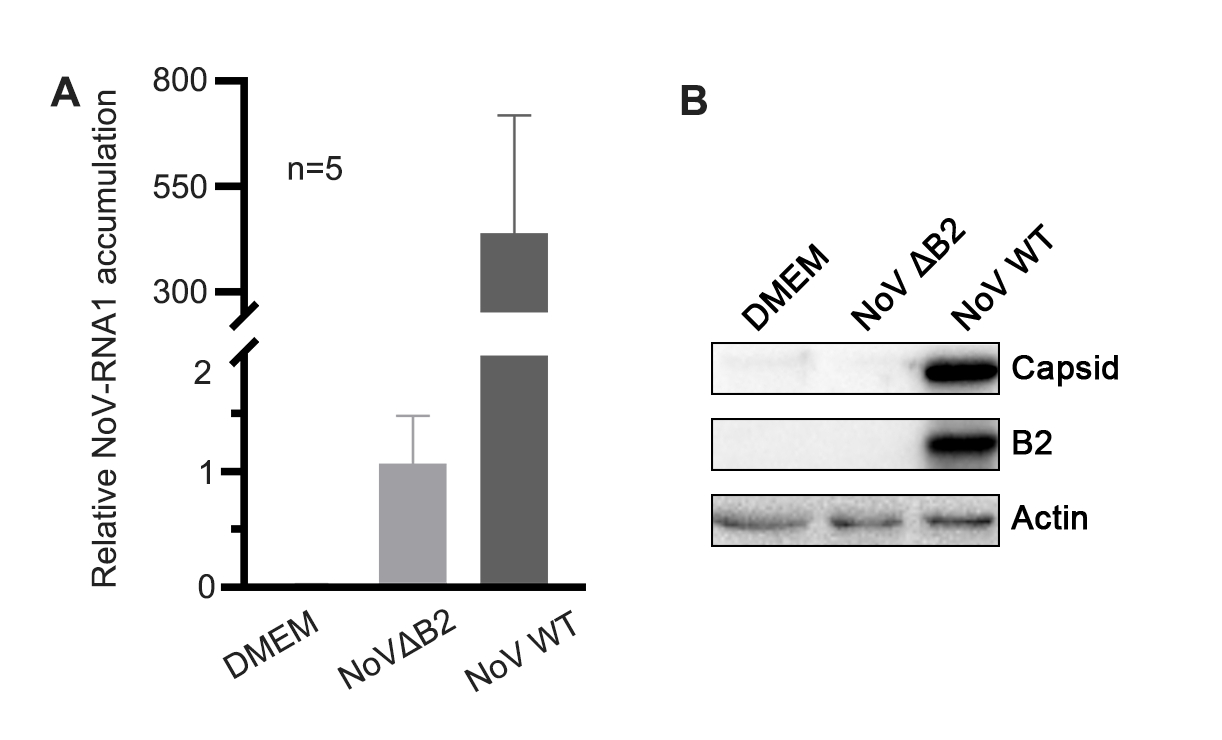

Supplement: S2 Fig — A. RNA1 accumulation level of WT NoV or NoVΔB2 measured by RT-qPCR from hind limb of C57BL/6 suckling mice at 7 dpi (n = 5 per group). The RNA1 level of NoVΔB2 infected C57BL/6 suckling mice was set as 1. B. Expression level of NoV capsid and B2 protein measured by Western blotting. Actin were used as a loading control. (TIF) [file ppat.1009790.s002.tif]

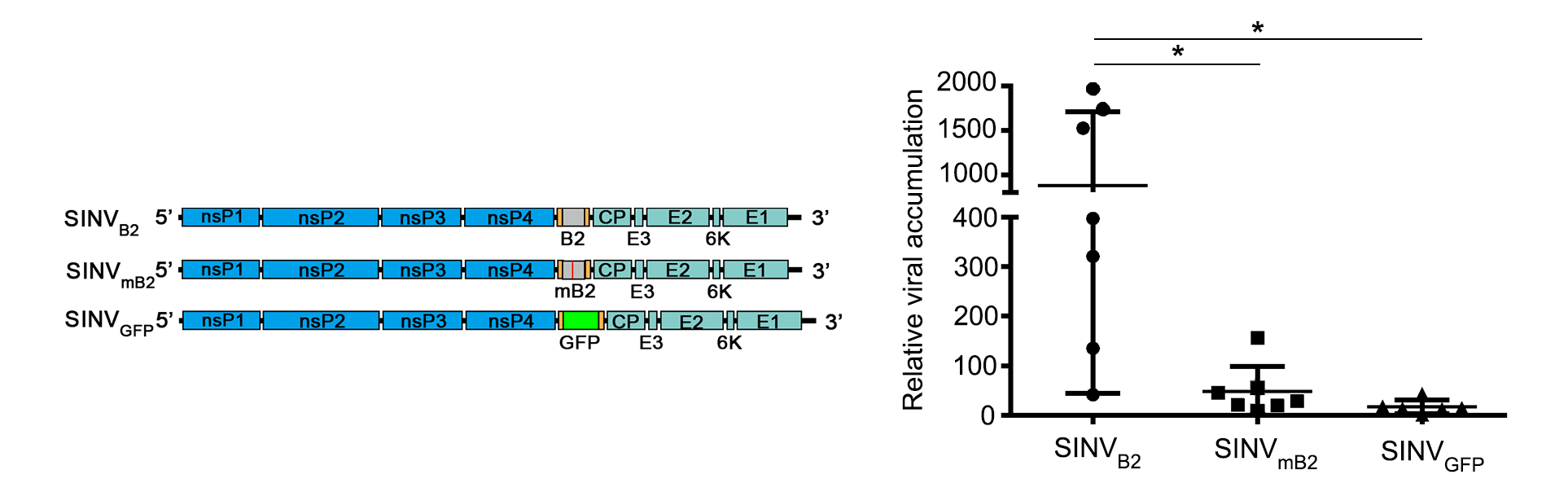

Supplement: S3 Fig — The genomic structure of SINVB2, SINVmB2 or SINVGFP (right) and relative viral accumulation determined by RT-qPCR from hindlimb of BALB/c suckling mice infected with SINVB2, SINVmB2 or SINVGFP at 3dpi. n = 6~7 per group. Error bars represent SD. * indicates p<0.05 (Student’s t-test). The viral RNA accumulation of SINVGFP was set as 1. (TIF) [file ppat.1009790.s003.tif]

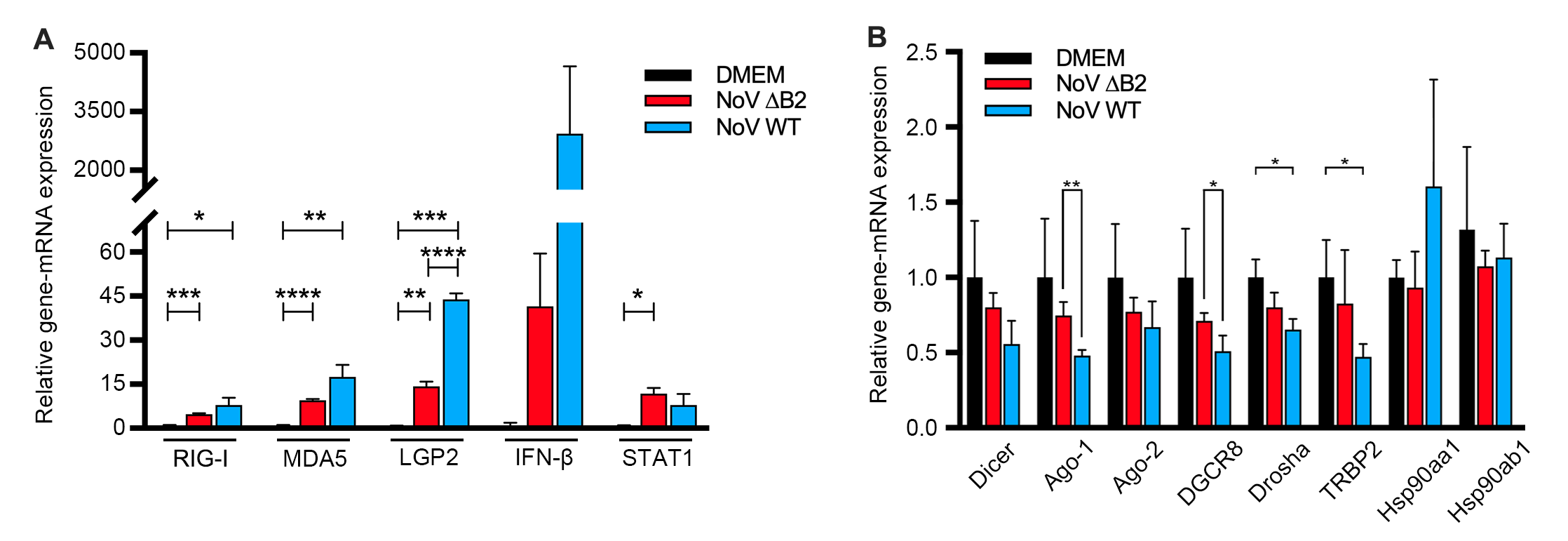

Supplement: S4 Fig — A, B. Expression levels of IFN (A) and RNAi (B) related genes in C57BL/6 suckling mice with NoV infection at 7dpi. All data were measured as the mean ± SD of three independent experiments. Asterisks indicate a significant difference level compared to control (Student’s t-test, *p<0.05, **p<0.01, ***p<0.001, ****p<0.0001). (TIF) [file ppat.1009790.s004.tif]

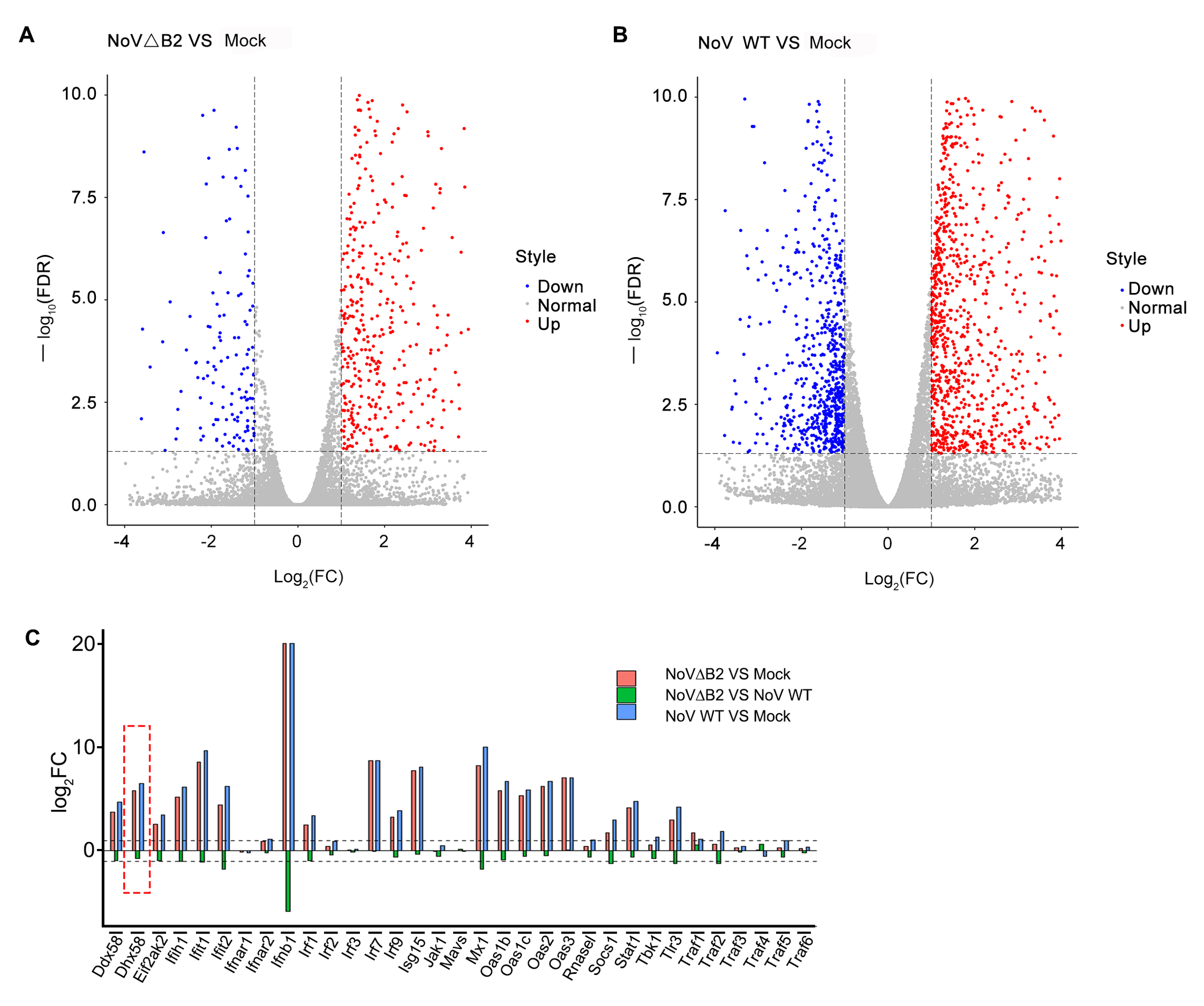

Supplement: S5 Fig — A, B. Volcano plots showing demonstrate false discovery rate (FDR) and fold-change (FC) of gene expression levels determined by RNAseq comparing BALB/c suckling mice inoculated by NoVΔB2 vs Mock (A), WT NoV vs Mock (B). Genes up-regulated (fold change > 2 and FDR< 0.05) are indicated in red and those down-regulated (fold change < 0.5 and FDR< 0.05) are indicated in blue. C. Differential expression of IFN pathway related genes from mRNA-seq data of NoV or NoVΔB2 inoculated BALB/c suckling mice at 3 dpi. Fold changes (FC) of 2 or 0.5 (|log2FC| = 1) are indicated by dotted lines. The log2FC was taken as 20 when the ratio of experimental group and mock group tended to be infinite due to a small denominator. (TIF) [file ppat.1009790.s005.tif]

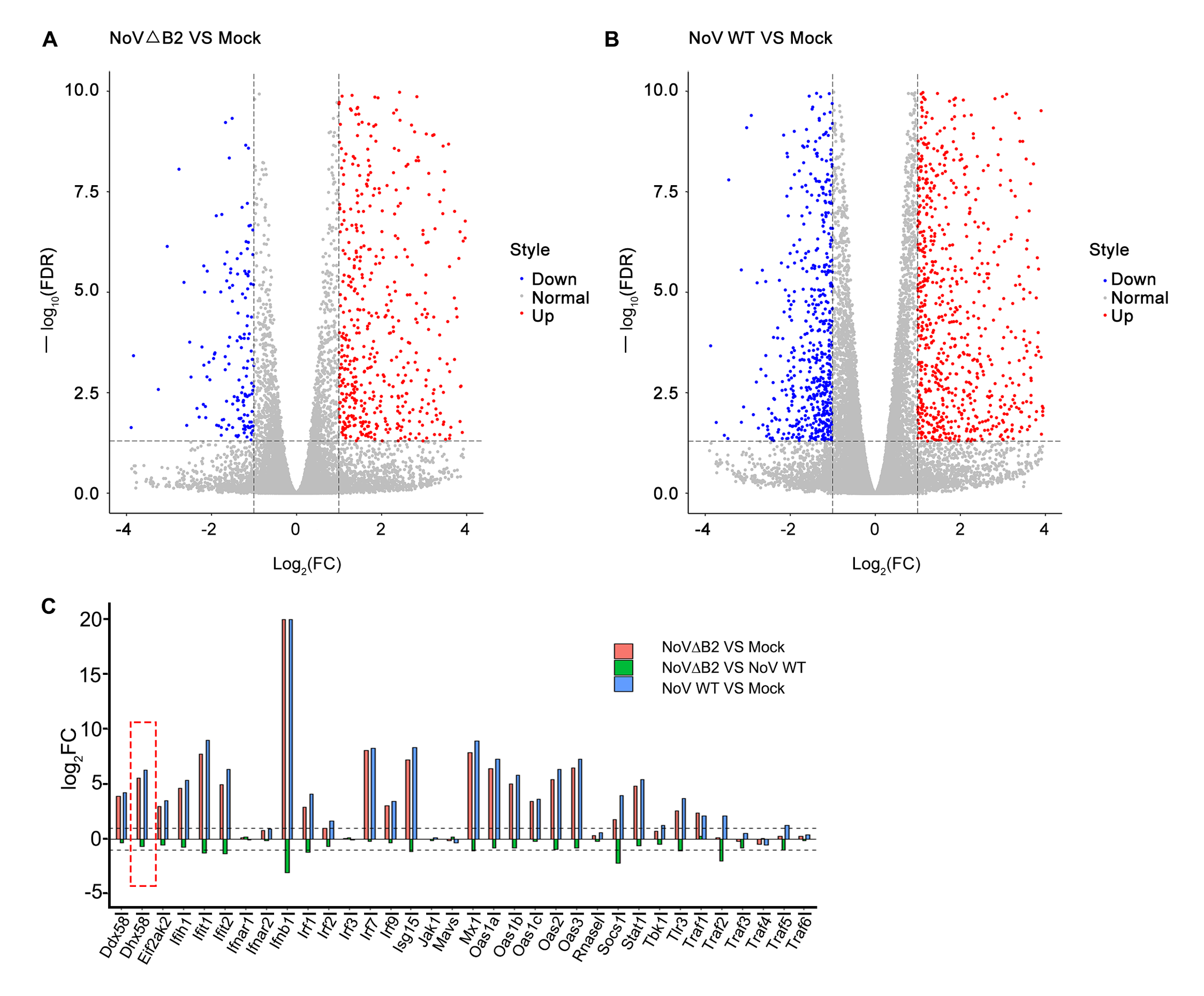

Supplement: S6 Fig — A, B. Volcano plots showing demonstrate false discovery rate (FDR) and fold-change (FC) of gene expression levels determined by RNAseq comparing C57BL/6 suckling mice inoculated by NoVΔB2 vs Mock (A), WT NoV vs Mock (B). Genes up-regulated (fold change > 2 and FDR< 0.05) are indicated in red and those down-regulated (fold change < 0.5 and FDR< 0.05) are indicated in blue. C. Differential expression of IFN pathway related genes from mRNA-seq data of NoV or NoVΔB2 inoculated C57BL/6 suckling mice at 3 dpi. Fold changes (FC) of 2 or 0.5 (|log2FC| = 1) are indicated by dotted lines. The log2FC was taken as 20 when the ratio of experimental group and mock group tended to be infinite due to a small denominator. (TIF) [file ppat.1009790.s006.tif]
