# Supplementary material for: Efficient Dicer processing of virus-derived double-stranded RNAs and its modulation by RIG-I-like receptor LGP2
Source: PLoS Pathog. 2021 Aug 3;17(8):e1009790. doi: 10.1371/journal.ppat.1009790 (PMC8362961; doi:10.1371/journal.ppat.1009790)
Supplement: S1 Table — (DOCX) [file ppat.1009790.s007.docx]

**S1 Table:** Contents and properties of the small RNA libraries sequenced

| Library | Total reads  (18-28nt) | miRNA^1^  (mature) | Virus reads  (18-28nt) | Virus reads of 21- to 23-nt | | | |
| --- | --- | --- | --- | --- | --- | --- | --- |
|  |  |  |  | Reads | % of miRNA | % of all sizes | (+)-strand % |
| PR8ΔNS1: 293T-Dicer KO +hDicer | 9,108,277 | 207,754 | 768,765 | 684,571 | 329.50% | 89.00% | 32.40% |
| PR8ΔNS1: 293T-Dicer KO +hDicer +IAV NS1 | 13,141,437 | 318,044 | 45,121 | 26,111 | 8.20% | 57.90% | 30.90% |
| PR8ΔNS1: 293T-Dicer KO +hDicer +ZIKV NS1 | 7,902,639 | 201,390 | 378,680 | 334,703 | 166.20% | 88.39% | 31.61% |
| NoV∆B2：suckling BALB/c 3dpi | 7,538,537 | 1,420,685 | 29,189 | 25,080 | 1.80% | 85.90% | 45.00% |
| NoV∆B2：Ago-IP(suckling BALB/c 3dpi) | 60,697,364 | 21,568,054 | 107,790 | 100,047 | 0.50% | 92.80% | 49.70% |
| NoV：suckling BALB/c 3dpi | 18,144,911 | 5,872,578 | 1,019,465 | 338,716 | 5.80% | 33.20% | 88.60% |
| NoV：Ago-IP(suckling BALB/c 3dpi) | 88,953,631 | 31,799,283 | 10,636 | 5,601 | 0.02% | 52.70% | 70.90% |
| NoV：B2-IP(suckling BALB/c 3dpi) | 97,845 | 13,160 | 31,197 | 16,866 | 128.20% | 54.10% | 40.30% |
| NoV∆B2: suckling C57BL/6 4dpi | 12,672,796 | 3,398,369 | 41,219 | 34,311 | 1.00% | 83.20% | 38.10% |
| NoV∆B2: suckling C57BL/6 7dpi | 19,643,646 | 10,489,173 | 22,945 | 21,095 | 0.20% | 91.90% | 33.90% |
| NoV: suckling C57BL/6 4dpi | 17,362,215 | 7,824,183 | 1,220,938 | 477,116 | 6.10% | 39.10% | 85.70% |
| NoV: suckling C57BL/6 7dpi | 5,974,094 | 2,338,381 | 301,420 | 166,347 | 7.10% | 55.20% | 59.70% |
| NoV: B2-IP(suckling C57BL/6 7dpi) | 7,056,582 | 652,831 | 1,151,786 | 541,170 | 82.90% | 47.00% | 58.10% |
| NoV: Ago-IP(suckling C57BL/6 7dpi) | 13,346,600 | 10,384,250 | 15,667 | 13,641 | 0.10% | 87.10% | 50.00% |
| SINV: Total(suckling BALB/c 3dpi) | 25,419,556 | 12,944,228 | 22,918 | 17,553 | 0.1% | 76.6% | 64.3% |
| SINV_B2_: Total(suckling BALB/c 3dpi) | 19,858,703 | 10,143,975 | 1,723 | 615 | 0.01% | 35.7% | 86.7% |
| SINV_mB2_: Total(suckling BALB/c 3dpi) | 21,166,041 | 10,733,024 | 470 | 396 | 0.004% | 84.3% | 48.5% |
| NoV∆B2: suckling C57BL/6 Lgp2^-/-^ 4dpi | 23,329,547 | 6,413,641 | 205,140 | 175,953 | 2.70% | 85.80% | 44.90% |
| NoV: suckling C57BL/6 Lgp2^-/-^ 4dpi | 15,047,306 | 3,693,351 | 516,496 | 237,968 | 6.40% | 46.10% | 62.60% |

^1^ Indicating the reads perfectly identical to mature microRNAs
