# Supplementary material for: Efficient Dicer processing of virus-derived double-stranded RNAs and its modulation by RIG-I-like receptor LGP2
Source: PLoS Pathog. 2021 Aug 3;17(8):e1009790. doi: 10.1371/journal.ppat.1009790 (PMC8362961; doi:10.1371/journal.ppat.1009790)
Supplement: S2 Table — (DOCX) [file ppat.1009790.s008.docx]

**S2 Table:** Primers, Related to Experimental Procedures

| **Primers for RT-qPCR** |  |
| --- | --- |
| **Name** | **Sequence (5’ to 3’)** |
|  |  |
| hActin mRNA-forward | ACCAACTGGGACGACATGGAGAAA |
| hActin mRNA-reverse | TAGCACAGCCTGGATAGCAACGTA |
| hIFN-β mRNA-forward | TGGGAGGCTTGAATACTGCCTCAA |
| hIFN-β mRNA-reverse | TCTCATAGATGGTCAATGCGGCGT |
| hRIG-I mRNA-forward | AAACCAGAGGCAGAGGAAGAGCAA |
| hRIG-I mRNA-reverse | TCGTCCCATGTCTGAAGGCGTAAA |
| hMAVS mRNA-forward | GGCCTGTGTCTCCATCTGTC |
| hMAVS mRNA-reverse | CCAGGGGATGAGGAGGAGAA |
| NoV RNA1 mRNA-forward | GTGTCGGAGTCCCCTATTCA |
| NoV RNA1 mRNA-reverse | ATGGTCGGTTCGTTCTTCAC |
| SINV nsP2 mRNA-forward | GGCGTTTCGCACTAAGAAAG |
| SINV nsP2 mRNA-reverse | TGCAGCAGTTTTTCCTCCTT |
| mActin mRNA-forward | ATTGGCAACGAGCGGTTCC |
| mActin mRNA-reverse | AGCACTGTGTTGGCATAGAGG |
| mRIG-I mRNA-forward | GAGAGTCACGGGACCCACT |
| mRIG-I mRNA-reverse | CGGTCTTAGCATCTCCAACG |
| mMDA5 mRNA-forward | TGATGCACTATTCCAAGAACTAACA |
| mMDA5 mRNA-reverse | TCTGTGAGACGAGTTAGCCAAG |
| mIFN-β mRNA-forward | CACAGCCCTCTCCATCAACTA |
| mIFN-β mRNA-reverse | CATTTCCGAATGTTCGTCCT |
| mSTAT1 mRNA-forward | AAATGTGAAGGATCAAGTCATGTG |
| mSTAT1 mRNA-reverse | CATCTTGTAATTCTTCTAGGGTCTTGA |
| mDicer mRNA-forward | GACCGTGTTCCTCGTCAACTCTG |
| mDicer mRNA-reverse | TCAAAACAGTCAAGGCGACATAGC |
| mAgo-1 mRNA-forward | TCGGAAGATTTCCAAGGATG |
| mAgo-1 mRNA-reverse | GTTGCCATTCCCAAGAGTGT |
| mAgo-2 mRNA-forward | AAGTCGGACAGGAGCAGAAA |
| mAgo-2 mRNA-reverse | GAAACTTGCACTTCGCATCA |

| **Name** | **Sequence (5’ to 3’)** |
| --- | --- |
|  |  |
| mDGCR8 mRNA-forward | GCTGCAGGAGTAAGGACAGG |
| mDGCR8 mRNA-reverse | TCGAGCACTGCATACTCCAC |
| mDrosha mRNA-forward | GGACCATCACGAAGGACACT |
| mDrosha mRNA-reverse | GATGTACAGCGCTGCGATAA |
| mTRBP2 mRNA-forward | CTTCCAAAAAGCTGGCAAAG |
| mTRBP2 mRNA-reverse | GAGCAACTGCGAAGGGATAG |
| mHsp90aa1 mRNA-forward | GTGTGCAACAGCTGAAGGAA |
| mHsp90aa1 mRNA-reverse | CTCTCCATGTTTGCTGTCCA |
| mHsp90ab1 mRNA-forward | GCGGCAAAGACAAGAAAAAG |
| mHsp90ab1 mRNA-reverse | CAAGTGGTCCTCCCAGTCAT |
| mLGP2 mRNA-forward | CCACGACCTGCTCATCTGTA |
| mLGP2 mRNA-reverse | GTACCGGCTCAAGATGGTGT |

| **Primers used for constructing plasmids** | |  |  |
| --- | --- | --- | --- |
| **Description** | **Name** | **Sequence (5’ to 3’)** |  |
| To clone NoV RNA1(g) (2767-2959) into pmirGLO plasmid | F1 | AGTAAAGCGGCCATCAAAGCTTAGCTAATCAAGTCACTTCCAGC |  |
|  | F2 | GTCGACGGTATCGATAAAGCTTTGCTGCGACTCGCGGTTTGATG |  |
| To clone NoV RNA1(ag) (2767-2959) into pmirGLO plasmid | F3 | GCTCGCTAGCCTCGAGTCTAGACTGCTGCGACTCGCGGT |  |
|  | F4 | CCTGCAGGTCGACTCTAGAGAGCTAATCAAGTCACT |  |

| **Primers used for generating dsRNA** | | | |
| --- | --- | --- | --- |
| **Description** | **Name** | **Sequence(5’ to 3’)** |  |
| To generate (+)-sense RNA GFP PCR products | F1 | GAAATTAATACGACTCACTATAGGGAGAATGGTGAGCAAGGGCG |  |
|  | F2 | AAGCACTGCACGCCGTAGG |  |
| To generate (-)-sense RNA GFP PCR products | F3 | GAAATTAATACGACTCACTATAGGGAGAAAGCACTGCACGCCG |  |
|  | F4 | ATGGTGAGCAAGGGCGAGGAGC |  |
